# Significance of SUMOylation in breast cancer progression: a comprehensive investigation using single-cell analysis and bioinformatics

**DOI:** 10.3389/fimmu.2025.1675874

**Published:** 2025-11-20

**Authors:** Wenxing He, Zhengkui Sun, Dongmei Li, Tenghua Yu

**Affiliations:** 1 Breast Cancer Center, Jiangxi Cancer Hospital (The Second Affiliated Hospital of Nanchang Medical College), Nanchang, China; 2 Jiangxi Key Laboratory of Translational Research for Cancer, Jiangxi Cancer Hospital (The Second Affiliated Hospital of Nanchang Medical College), Nanchang, China

**Keywords:** breast cancer, SUMOylation, biomarkers, immune infiltration, scRNA

## Abstract

**Background:**

Breast cancer remains a major global health challenge because of limitations in early detection and therapeutic outcomes. This study employed bulk and single-cell RNA sequencing to investigate SUMOylation-associated molecular networks, aiming to identify prognostic biomarkers and potential therapeutic applications.

**Methods:**

Transcriptomic profiling was performed on 1,445 breast cancer and 113 normal samples to identify differentially expressed genes. Four hub genes, *NR3C2*, *CDCA8*, *AURKA*, and *PLK1*, were prioritized using machine learning. Consensus clustering stratified patients into molecular subtypes based on the hub gene expression patterns. Differential immune infiltration analysis was used to evaluate 28 immune cell populations between the subtypes. Hub gene-immune cell interactions were visualized using bubble diagrams. Pharmacogenomic sensitivity profiling was performed using subtype-specific drug response data. Single-cell sequencing identified epithelial subclusters enriched for hub genes, and transcription factor networks were analyzed using SCENIC. Pan-cancer validation was performed to assess the oncogenic role of hub genes in 21 malignancies. Statistical significance was determined using the Student’s *t*-test (*p* < 0.0001).

**Results:**

Tumor tissues exhibited significant upregulation of *CDCA8*, *AURKA*, and *PLK1*, whereas *NR3C2* was notably downregulated (*p* < 0.0001). Consensus clustering identified two distinct molecular subtypes: Subtype1, characterized by *NR3C2* upregulation and poorer prognosis, and Subtype2, distinguished by enhanced expression of *CDCA8*, *AURKA*, and *PLK1*, correlating with favorable outcomes. Notably, PIK3CA mutations were prevalent in Subtype1, whereas *TP53* mutations dominated Subtype2. Immune infiltration profiles differed significantly between the two subtypes for most immune cell types. Pharmacogenomic assessments revealed distinct drug sensitivity profiles for each subtype in response to various therapeutic agents. A pan-cancer analysis of the four hub genes demonstrated consistent expression patterns, immune correlations, and prognostic associations across malignancies.

**Conclusion:**

Our findings reveal that SUMOylation subtypes in breast cancer exhibit distinct prognostic, immunological and pharmacogenomic profiles. These insights may provide candidate biomarkers for future personalized treatment strategies for breast cancer and potentially for other malignancies.

## 1 Introduction

Breast cancer (BRCA) is a major malignancy affecting women globally, imposing substantial multifaceted challenges, including physical and psychological distress and socioeconomic challenges, particularly concerning healthcare expenditures and long-term care costs (1). Epidemiological data reveal that BRCA is the most prevalent form of cancer among women, accounting for 11.7% of all cancer cases and serving as the leading cause of cancer-related mortality in this population (2). Current clinical interventions for BRCA include surgical resection, radiation therapy, and systemic pharmacological regimens (chemotherapy and targeted therapies). However, these approaches have persistent limitations in terms of early detection precision, therapeutic personalization, and drug resistance management (3, 4). This study addresses the critical unmet needs by systematically exploring novel molecular biomarkers and therapeutic targets to enhance diagnostic accuracy and optimize treatment paradigms in breast oncology.

In this study, we investigated the role of SUMOylation (small ubiquitin-like modifier) in breast carcinogenesis. As a crucial post-translational modification, SUMOylation modulates the expression of both oncogenic and tumor suppressor genes, thereby regulating fundamental cellular activities, including gene expression, cell cycle progression, stress responses (5), and epithelial–mesenchymal transition (EMT) (6–8). SUMOylation is pivotal in tumor EMT, metastasis, and resistance to therapy (9). Emerging data implicate SUMOylation in immune evasion mechanisms by influencing immune cell functionality within the tumor microenvironment (TME) (10–12). Its specific roles in mitotic regulation, transcriptional control, and DNA damage response make it a high-value focus for breast cancer research (13–15).

**Figure 1:**
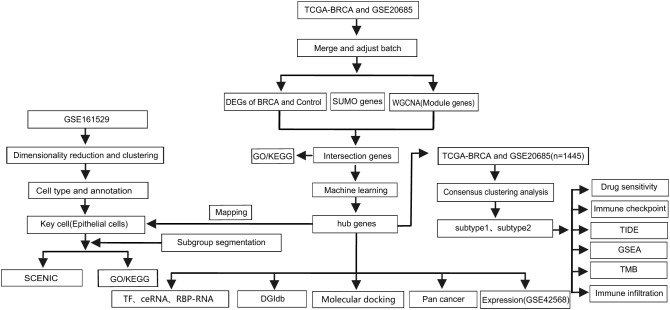
Research flowchart. BRCA, Breast cancer; ceRNAs, competing endogenous RNAs; DEGs, differentially expressed genes; DGIdb, the Drug-Gene Interaction Database; GSEA, Gene Set Enrichment Analysis; GO, Gene Ontology; KEGG, Kyoto Encyclopedia of Genes and Genomes; TIDE, Tumor Immune Dysfunction and Exclusion; RBP, RNA binding protein; SCENIC, Single-Cell Regulatory Network Inference and Clustering; TF, transcription factor; TMB, tumor mutation burden.

Despite this progress, the integrated bulk and single-cell RNA sequencing characterization of SUMOylation networks, their clinical relevance to BRCA heterogeneity, and their translational potential for immune microenvironment modulation remain underexplored. Therefore, further study of SUMOylation in BRCA will not only elucidate the molecular mechanisms but may also offer new targets and strategies for the diagnosis and management of BRCA. To address these challenges, this study focused on a specific molecular process, SUMOylation. We employed an integrative bioinformatics framework combining bulk transcriptomic profiling (16), single-cell RNA sequencing (scRNA-seq) (17), and weighted gene co-expression network analysis (WGCNA) (18) with machine learning models (19) to decode SUMOylation-associated molecular networks. The strength of this study lies in the integration of bulk and single-cell RNA sequencing data, combined with computational approaches, to investigate breast carcinogenesis with a focus on SUMOylation. Our primary objective was to identify hub genes associated with SUMOylation and their roles in mammary tumorigenesis, laying the groundwork for early diagnostic biomarkers and personalized therapies for BRCA. The flowchart of this study is shown in **Figure 1**.

## 2 Materials and methods

### 2.1 Acquisition and analysis of routine transcriptome data

All datasets employed in this study were publicly available and obtained from the Gene Expression Omnibus (GEO; https://www.ncbi.nlm.nih.gov/geo/) and The Cancer Genome Atlas (TCGA; https://portal.gdc.cancer.gov/) databases. Initially, the datasets GSE20685 and GSE42568 were acquired from the GEO database using the R package “GEOquery” (v2.62.2). GSE20685, sequenced on the Affymetrix U133 Plus 2.0 platform (HG-U133_Plus_2 array, GPL570), comprised 327 BRCA samples with complete survival information. GSE42568, also sequenced on the GPL570 platform, contained 121 samples, including 17 normal breast tissue controls and 104 BRCA samples. Additionally, whole-genome expression profile data in TPM format and clinical data for BRCA tissues were retrieved from TCGA using the bioinformatics toolkit TCGAbiolinks (v2.25.0) in the R environment (16). The TCGA-BRCA dataset encompassed 1231 samples, with 1118 tumor samples (BRCA) and 113 control samples (Control).

Various analytical sets were constructed from these data to satisfy specific requirements. Particularly, 1118 BRCA samples with complete clinical information and 113 control samples from TCGA-BRCA (n = 1231) were merged with 327 BRCA samples with complete clinical information from GSE20685 (n = 327), resulting in a training set of 1445 BRCA samples and 113 control samples. This dataset was used for all analyses except single-cell analysis (where only the 1445 BRCA samples were used for consensus clustering analysis). The 104 BRCA samples and 17 control samples from GSE42568 (n = 121) were used for external validation to assess the generalizability of the results. The ComBat method from the R package “sva” (v3.42.0) was utilized to correct for batch effects induced by non-biological technical variations (20) (**Supplementary Figure S1**). The correction efficacy was examined using principal component analysis (PCA). This study followed all relevant data usage protocols established by the respective repositories. From the GeneCards database (https://www.genecards.org/), 511 SUMOylation-related genes were identified (**Supplementary Table S1**).

### 2.2 Acquisition and computational analysis of single-cell sequencing datasets

The GEO functions as a primary repository for the deposition and retrieval of single-cell sequencing data, encompassing various experimental designs and tissue-specific profiles. We retrieved the scRNA-seq dataset GSE161529 from the NCBI GEO database to investigate BRCA heterogeneity at the single-cell resolution. The GSE161529 dataset, sequenced on the GPL18573 Illumina NextSeq 500 (*Homo sapiens*) platform, comprised 13 samples annotated as normal controls and 38 tumor samples from patients with adenocarcinoma as disease samples, all of which were included in this study. Initially, low-quality cells and genes were excluded based on the following specific criteria: (1) cells with a gene expression range between 200 and 7,000; (2) cells with unique molecular identifier counts below 75,000; (3) cells with a mitochondrial gene percentage of less than 25%; and (4) cells with a gene-to-read count ratio exceeding 0.7. Data normalization was conducted using the “normalizedata” function found within the Seurat R package. Following this normalization, highly variable genes within single cells were identified by evaluating the balance between the average expression levels and their corresponding dispersion. Subsequently, PCA was performed, with significant principal components (PCs) serving as the basis for graph-based clustering. The Harmony method was applied to address and mitigate batch effects present across the various samples. For clustering, we used the FindClusters function, which is based on a clustering algorithm optimized for shared nearest neighbor modularity, generating 27 clusters on 15 PCs with a resolution of 0.6. Uniform Manifold Approximation and Projection (UMAP) was implemented using the “RunUMAP” algorithm. UMAP-1 and UMAP-2 were used to demonstrate the cell clustering. To identify differentially expressed genes (DEGs) across distinct cellular subpopulations, we performed a computational analysis using the default parameters set by Seurat on normalized transcriptomic datasets. Following the identification of cellular clusters using type-specific molecular markers (21), a quantitative assessment of cellular subtype distribution was conducted.

### 2.3 Analysis of regulatory networks at the single-cell level (transcription factors)

We conducted a cis-regulatory analysis using pySCENIC (v0.11.2) to identify the key transcription factors (TFs) across various cell types (22). This tool infers gene regulatory networks by examining co-expression patterns and performing DNA motif analysis. Subsequently, the network activity for each cell type was evaluated by calculating the area under the curve (AUC).

Briefly, we used GENIE3 to identify TFs and assemble them into modules (rules), followed by gene-motif enrichment analysis using RcisTarget, focusing on the regions 500 base pairs upstream to 100 base pairs downstream of the transcription start site. Subsequently, we assessed the activity of the rules for each individual cell within the dataset using AUCell. Ultimately, we visualized the activity of the binarized regulatory subnetwork using a tSNE plot. TFs corrected by the Benjamini–Hochberg false discovery rate (FDR) < 0.05 were considered for further investigation. Subsequently, we applied Pearson’s correlation analysis to quantify the association between the rules and IFN-I scores.

### 2.4 Network-based co-expression profiling using weighted correlation and functional module detection

Based on the training set, a co-expression network was established using WGCNA, employing V1.70–3 of the corresponding R package (18). To construct a biologically meaningful scale-free network, pairwise gene expression similarities were quantified using Pearson’s correlation analysis. This was followed by a power function transformation of the resulting correlation coefficients for the network edge weighting. The weighted adjacency matrix was constructed by applying a power transformation (β = 5) to the co-expression similarity measures using the R package “PickSoftThreshold.” Gene modules, representing highly interconnected clusters of genes with coordinated expression patterns, were identified through hierarchical clustering in WGCNA, with color coding used for visual differentiation of the modules. The dynamic tree cut algorithm was implemented for module detection in network analysis. During the module identification phase, the adjacency matrix (which quantifies topological similarity) was transformed into a topological overlap matrix). Subsequent module recognition was achieved using hierarchical clustering analysis. Pearson correlations between module eigengenes (MEs, the first PCs) and SUMOylation-related genes were analyzed to identify module–SUMOylation associations. Modules significantly associated with senescence-related genes were identified using network analysis. Gene co-expression patterns were illustrated using topological overlap heatmaps to characterize network architecture. Module interactions were subsequently analyzed by generating two complementary representations: a hierarchical dendrogram of eigengene relationships and corresponding correlation heatmap.

### 2.5 Consensus clustering analysis

The BRCA samples (n = 1,445) in the training cohort were stratified into molecular subtypes through consensus clustering analysis of SUMOylation-associated gene expression patterns, performed using the R package ConsensusClusterPlus (v1.58.0) (23). The clustering procedure was iterated 1000 times with k = 6 to ensure robust stability in pattern identification.

### 2.6 Machine learning

Feature selection and model optimization were performed using three complementary methods. The Support Vector Machine–Recursive Feature Elimination (SVM-RFE) algorithm, implemented via the e1071 R package, systematically reduced feature dimensionality through recursive elimination of low-weight features using a linear kernel, with 10-fold cross-validation. Linear model optimization with variable retention was conducted through Least Absolute Shrinkage and Selection Operator (LASSO) regression via the *glmnet* package (v4.1-4), applying L1 regularization under a binomial distribution. The optimal regularization parameter λ was selected as λ.min through 10-fold cross-validation. Random forest analysis was carried out using the *randomForest* package, with ntree set to 500 and mtry tuned to minimize out-of-bag error. The mtry value, which represents the number of variables randomly sampled as candidates at each split, was determined using the minimum error. Additionally, the ntree value, which indicates the number of trees to be cultivated within the forest, was selected based on the image value that exhibited stability. Using mean decrease in accuracy (MDA) and mean decrease in Gini index (MDG) as feature importance criteria, we identified the top 10 DEGs through random forest analysis. Subsequently, by integrating the results from SVM-RFE, LASSO, and random forest, the intersection of genes identified by all three algorithms was taken, yielding the robust hub genes for further investigation.

### 2.7 Differential analysis

We utilized the FindAllMarkers function from the “Seurat” R package with default settings, specifically |log_2_Fold Change(log_2_FC)| > 0.25 and an adjusted *p*-value (*adj.p*) < 0.05, to identify DEGs between key cell types and other cell groups. To identify DEGs between BRCA tissues and their corresponding healthy control samples, as well as among various disease subtypes, we used the “limma” R package (v3.50.0) with screening criteria of |log_2_FC| > 1 and *adj.p* < 0.05.

### 2.8 Gene Ontology and Kyoto Encyclopedia of Genes and Genomes pathway enrichment analyses

The Gene Ontology (GO) (24) enrichment analysis systematically evaluates three principal ontological domains: biological processes (BP), molecular functions (MF), and cellular components (CC). Kyoto Encyclopedia of Genes and Genomes(KEGG) (25), a widely recognized biological database, enables systematic identification of dysregulated metabolic pathways associated with specific gene clusters. The overlapping gene set underwent GO and KEGG pathway enrichment analyses using the clusterProfiler R package (v4.2.2) (26), with statistical significance set at *p* < 0.05.

### 2.9 Gene Set Enrichment Analysis

Gene Set Enrichment Analysis (GSEA) (27), a computational methodology, evaluates whether predefined gene sets demonstrate statistically significant and concordant variations between distinct biological states. The R package “limma” (v3.50.0) (28) was used to conduct differential expression analysis comparing gene expression among subtypes identified through consensus clustering, resulting in subtype-specific fold change (FC) values. GSEA was performed with the clusterProfiler package (v4.2.2) using genes ranked by log_2_FC values.All statistical evaluations employed 1000 stochastic rearrangements of genetic clusters. The c2.cp.kegg. v7. 5.1. symbols gene set from the Molecular Signatures Database (MSigDB) served as the reference gene set (28–30). Gene sets exhibiting *p*-values below the 0.05 threshold were defined as statistically enriched.

### 2.10 Immune microenvironment profiling

The single-sample Gene Set Enrichment Analysis (ssGSEA), derived from the conventional GSEA methodology, was used to calculate individual enrichment scores for specific gene sets across separate biological samples (31). This method quantifies the level of coordinated activation or suppression of defined gene sets within individual samples, assigning specific scores to each sample–pathway combination.

Immune cell marker gene datasets were obtained from the Tumor–Immune System Interaction Database. The comprehensive collection encompassed major lymphocyte subsets and myeloid populations: (1) T cell subsets: activated, central memory, and effector memory populations in both CD8+ and CD4+ lineages, along with specialized T helper cells (follicular, γδ, Th1, Th2, and Th17) and regulatory T cells; (2) B cell lineages: activated, immature, and memory phenotypes; (3) natural killer populations: CD56bright, CD56dim, and NKT cells; (4) dendritic cell subsets: activated, plasmacytoid, and immature variants; and (5) innate immune components: macrophages, monocytes, neutrophils, eosinophils, mast cells, and myeloid-derived suppressor cells (32).

The gene expression profiles of individual samples were analyzed to obtain relative enrichment scores for distinct immune cell populations. To compare immune infiltration patterns across molecular subtypes, graphical representations were generated using the ggplot2 package (v3.3.6) in R (33).

### 2.11 Tumor Immune Dysfunction and Exclusion

The Tumor Immune Dysfunction and Exclusion (TIDE) computational framework (http://tide.dfci.harvard.edu) was used to evaluate immunotherapy responsiveness in clinical cohorts (34).

### 2.12 Immune checkpoints

A comparative analysis of key immune checkpoint genes was performed across the cohorts. These regulatory molecules modulate signaling modulators in immune cells, maintaining a balance of immune activation thresholds by preventing hyperimmune responses.

### 2.13 Tumor mutational burden quantification

Genomic variations were analyzed using the mutation data from 991 BRCA tissue samples. The MAF-compliant bioinformatics toolkit (v2.10.05) was used to characterize somatic alterations, including single nucleotide polymorphisms, insertions/deletions, tumor mutation burden (TMB), and mutation frequencies across different clusters (35). The 20 most frequently mutated genes (FMGs) were identified as key oncogenic drivers of malignancy progression (36).

### 2.14 Pharmacological responsiveness profiling

Therapeutic drug sensitivity across BRCA subtypes was evaluated using half-maximal inhibitory concentration (IC50) values and gene expression profiles from the Genomics of Drug Sensitivity in Cancer database (release 2022) (37). The oncoPredict algorithm (v0.2) was used for the computational modeling (38).

### 2.15 Chemogenomic interplay investigation

Prognostic genes were screened against the Drug–Gene Interaction Database (DGIdb; https://www.dgidb.org) (39) to identify existing agonists or inhibitors with the aim of repurposing approved BRCA therapeutics.

### 2.16 Pan-cancer data analysis

We conducted a comprehensive pan-cancer analysis of these key genes, assessing their transcriptional profiles in malignant tumors, prognostic implications, and their relationship with the characteristics of the immune microenvironment. Whole-genome expression and clinical data for 33 cancer types were retrieved from TCGA using the TCGAbiolinks package (v2.25.0) in R. We calculated the differences in gene expression between tumor and normal samples across the 33 cancer datasets from the TCGA database using the Wilcoxon test. For survival analysis, we used a univariate Cox regression model to evaluate the effect of prognostic gene expression on cancer prognosis. Finally, we computed the immune infiltration scores for all tumor samples using the ssGSEA algorithm and assessed the correlation between the immune infiltration scores and the expression levels of the prognostic genes.

### 2.17 RNA-binding protein–mRNA interactome modeling

This study explored ncRNA interactions via the StarBase platform (https://starbase.sysu.edu.cn/tutorialAPI.php#RBPTarget) using CLIP-seq, degradome-seq, and RNA–RNA interaction data to investigate the correlations between mRNA and RNA-binding protein (RBP) expression. In BRCA studies, we established significance thresholds (*p* < 0.05) and minimum cluster/clip-exposure thresholds (both ≥5) to identify biologically significant mRNA–RBP interactions. These validated pairs were subsequently visualized as interaction networks using Cytoscape software(v3.9.1).

### 2.18 TF interconnectivity profiling

TFs regulate gene expression by binding to DNA in a sequence-specific manner, thereby coordinating various cellular processes and developmental pathways. The TRRUST database (http://www.grnpedia.org/trrust/) provides curated transcriptional regulation data encompassing 8444 human TF–target interactions (800 TFs) and 6552 murine entries (828 TFs). This resource allows for the systematic identification of shared transcriptional regulators in functionally related gene clusters.

### 2.19 ceRNA network construction

To address the incomplete understanding of competing endogenous RNA (ceRNA) mechanisms in BRCA pathogenesis, we conducted reverse miRNA prediction for key genes using three validated databases: miRTarBase (https://mirtarbase.cuhk.edu.cn/-miRTarBase/miRTarBase_2022/php/index.php) (40), starBase 2.0 (https://starbase.sysu.edu.cn/starbase2/index.php) (41), and miRDB (https://mirdb.org/index.html). This endeavor sought to predict the lncRNAs that share miRNAs with these crucial genes, ultimately facilitating the construction of a ceRNA network.

### 2.20 Statistical analysis

Statistical analyses were conducted using R software (v4.1.2). We used Spearman’s rank correlation to evaluate the association between variables. Statistical comparisons between groups were performed using the Wilcoxon rank-sum test. Survival differences between molecular subtypes were compared using the Kaplan–Meier method and assessed with the log-rank test. Hazard ratios (HR) and 95% confidence intervals (CI) were calculated to quantify the magnitude of prognostic differences. Statistical significance was defined as a two-tailed *p*-value of < 0.05.

## 3 Results

### 3.1 Screening of the module most relevant to SUMOylation using WGCNA

**Figure 2:**
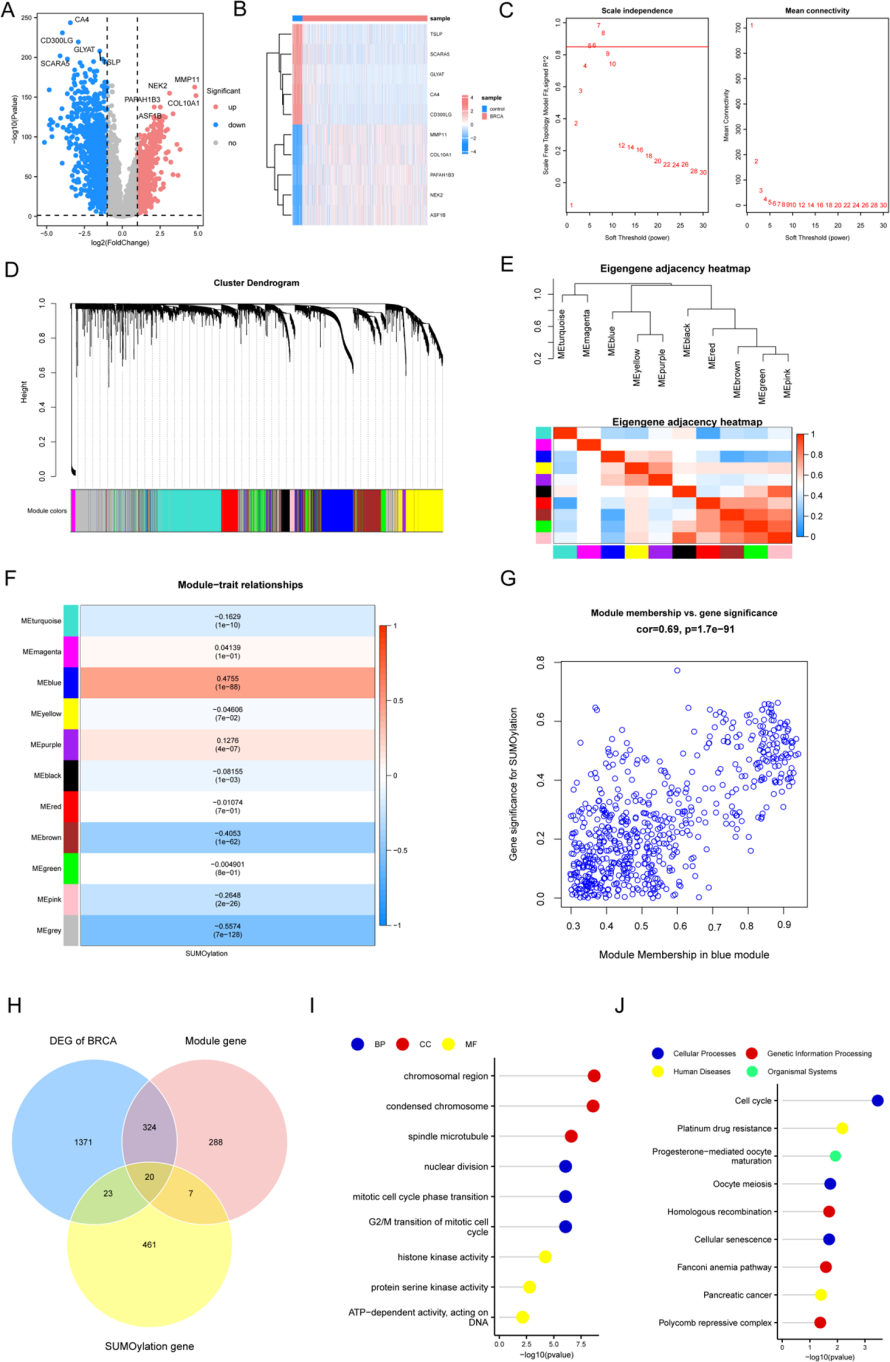
WGCNA-based characterization of co-expression modules correlated with SUMOylation modification. **(A)** Volcano plot illustrating DEGs in BRCA versus control specimens. **(B)** Heatmap displaying the five most significantly upregulated and downregulated DEGs across study cohorts. **(C) **Soft thresholding power plot showing the scale-free topology model fit index (R²) at β=5. **(D)** WGCNA revealing distinct modules of co-expressed data. **(E)** Module eigengene interaction heatmap with chromatic representation of inter-module correlations (red: high, blue: low), diagonal elements denoting meta-module relationships. **(F)** Consensus module-trait association matrix quantifying correlations between eigengenes and SUMOylation status, with color-coded coefficients and corresponding *p*-values. **(G)** Scatterplot demonstrating concordance between gene significance for SUMOylation and intramodular connectivity within the blue module (Cor = Pearson correlation coefficient). **(H)** Three-way Venn diagram depicting overlapping gene sets from differential expression analysis, SUMOylation-associated genes, and network modules. **(I)** GO enrichment analysis visualization using lollipop plot of the intersection genes. **(J)** KEGG pathway enrichment profile presented in lollipop plot format.

A comparative transcriptome analysis between BRCA and control samples revealed 1738 DEGs meeting statistical thresholds (|log_2_FC| > 1; FDR-*adj.p* < 0.05). In BRCA samples, 669 genes were upregulated and 1069 were downregulated compared to those in the controls (**Supplementary Table S2**).The differential expression profile is illustrated in a volcano plot (**Figure 2A**).Additionally, a heatmap was used to display the top five upregulated genes (*MMP11, NEK2, COL10A1, PAFAH1B3*, and *ASF1B)* and the five most significantly downregulated genes *(CA4, CD300LG, GLYAT, TSLP*, and *SCARA5*) ranked by *p*-value (**Figure 2B**). Subsequently, WGCNA identified the SUMOylation-associated gene modules. Scale independence and mean connectivity analyses demonstrated that a soft thresholding power of 5 (**Figure 2C**) achieved optimal network properties, with a mean connectivity approaching 0 and scale independence exceeding 0.85. Eleven co-expression modules were identified, excluding unrelated genes clustered in the gray module (**Figure 2D**). A heatmap depicting the eigengene network was used to examine inter-module connections and identify associated features (**Figure 2E**). To investigate the functional relevance of module-associated genes, we correlated the 11 MEs with SUMOylation phenotype genes and identified the key associations. The module–trait correlation heatmap (**Figure 2F**) revealed that the blue module (containing 639 genes) most accurately reflected SUMOylated protein modifications. A scatter plot of SUMOylation-related genes versus blue module membership values indicated a strong positive correlation (cor = 0.69, *p* < 0.05) (**Figure 2G**), suggesting that the key hub components within the blue module were strongly associated with SUMOylation-related gene characteristics. Subsequently, we identified 20 genes at the intersection of DEGs between the BRCA and control groups, blue module genes, and SUMOylation phenotype genes, and plotted a Venn diagram. These genes are potentially involved in BRCA pathogenesis and progression and their association with SUMOylation is illustrated in **Figure 2H**.

To explore the biological roles of the 20 overlapping genes, we performed Gene Ontology (GO) term enrichment (**Supplementary Table S3**) and Kyoto Encyclopedia of Genes and Genomes (KEGG) pathway analyses (**Supplementary Table S4**). GO analysis revealed significant enrichment in biological processes (BP), including nuclear division, G2/M phase transition, and mitotic cycle phase transitions; cellular components (CC), including chromosomal regions, condensed chromosomes, and spindle microtubules; and molecular functions (MF), including histone kinase, protein serine kinase, and ATP-dependent DNA-binding activities (**Figure 2I**).

KEGG pathway analysis (**Supplementary Table S4**) identified enrichment in cellular processes, including the cell cycle, oocyte meiosis, and cellular senescence; genetic information processing, including homologous recombination, the Fanconi anemia pathway, and polycomb repressive complex; human diseases, including platinum drug resistance and pancreatic cancer; and organismal systems, including progesterone-mediated oocyte maturation (**Figure 2J**).

### 3.2 Hub gene selection using machine learning algorithms

**Figure 3:**
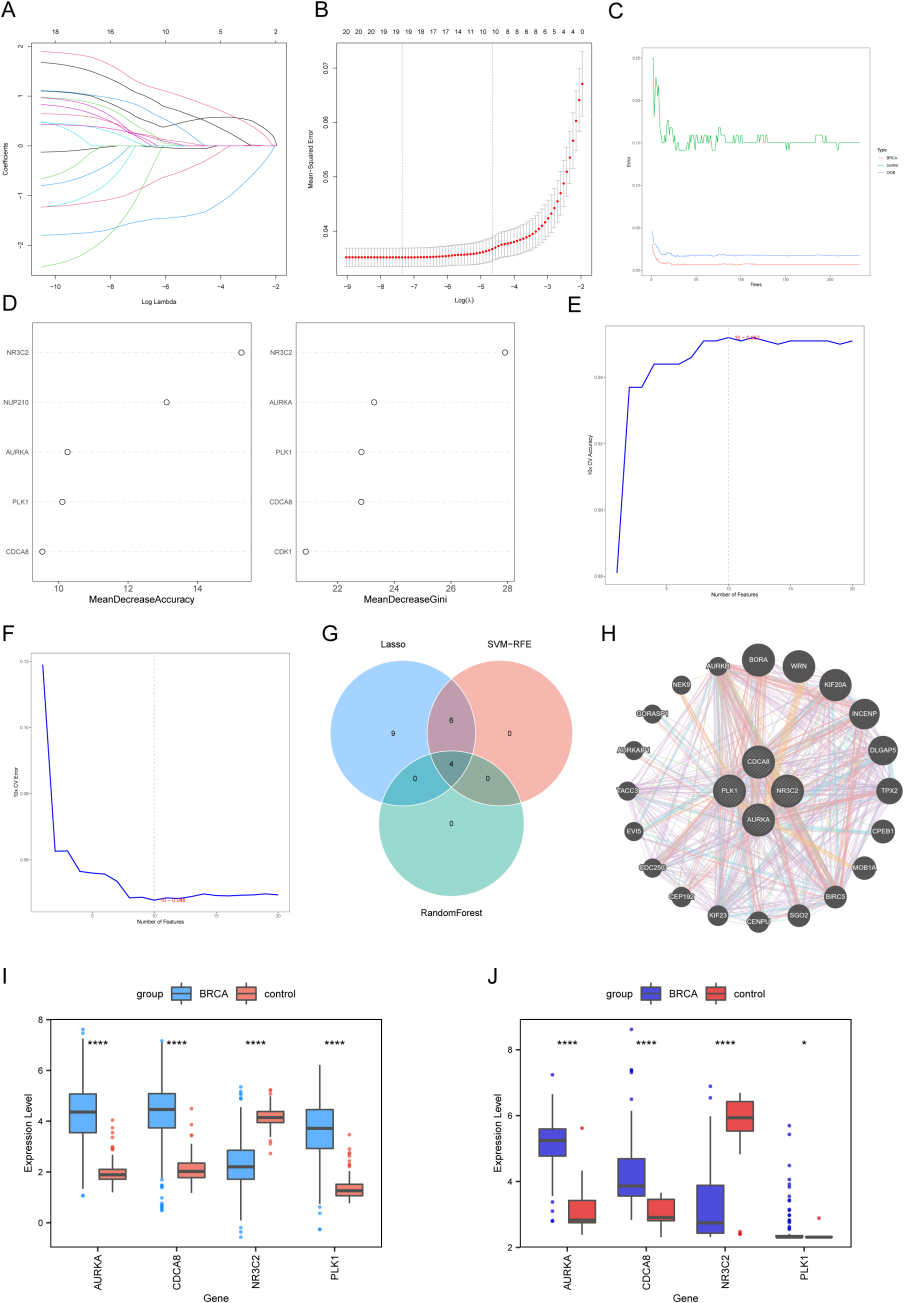
Screening of hub genes using machine learning methods. **(A)** The trace plot of the LASSO regression coefficients as a function of log(lambda), where the horizontal axis displays lambda’s logarithmic tuning parameter values while the vertical axis shows coefficients derived through independent calculations. **(B)** LASSO regression cross-validation curves for each lambda. **(C)** Comparison of the error rate of the Random Forest algorithm with the number of trees in the forest. **(D)** The top five phenotype-related DEGs ranked by two types of importance measures in the Random Forest algorithm. **(E)** Accuracy curve of the Support Vector Machine (SVM). **(F)** Error rate curve of the SVM. **(G)** Venn diagram showing the identification of hub genes. **(H)** Protein interaction network among hub genes. **(I)** Boxplot illustrating hub gene expression levels (BRCA vs. controls). **(J)** Box plots assessing hub gene expression profiles in BRCA vs. control cohorts using GSE42568. Statistical significance levels are denoted as follows: ****p < 0.0001, *p < 0.05.

To identify the most significant genes among the 20 overlapping genes, we applied LASSO regression, random forest, and SVM-RFE. LASSO regression identified 19 candidate genes (**Figures 3A, B**). The random forest method, using MDA and MDG feature weights, highlighted four key genetic markers from the top 5 candidate genes (**Figures 3C, D**). Using SVM-RFE analysis, we identified 10 key biomarkers (**Figures 3E, F**). The intersection of results from these three selection methods identified four central genes (*NR3C2*, *CDCA8*, *AURKA*, and *PLK1*) for further investigation (**Figure 3G**). Protein interaction analysis using GeneMANIA (https://genemania.org/) revealed functional associations between these core genes and 20 associated partners (**Figure 3H**).

Next, we evaluated the differences in hub gene expression between the BRCA and control groups. The four hub genes demonstrated significant differential expression between groups. The expression levels of pivotal regulatory genes (*CDCA8*, *AURKA*, and *PLK1*) were significantly higher in tumor samples than in control samples, whereas *NR3C2* was significantly downregulated in cancerous tissues (**Figure 3I**). Validation using the external dataset GSE42568 confirmed the consistent differential expression of these genes, which aligned with the training set findings. These findings underscore the potential role of these hub genes in BRCA pathogenesis and their possible therapeutic relevance (**Figure 3J**).

### 3.3 Hub gene-based subtyping analysis

**Figure 4:**
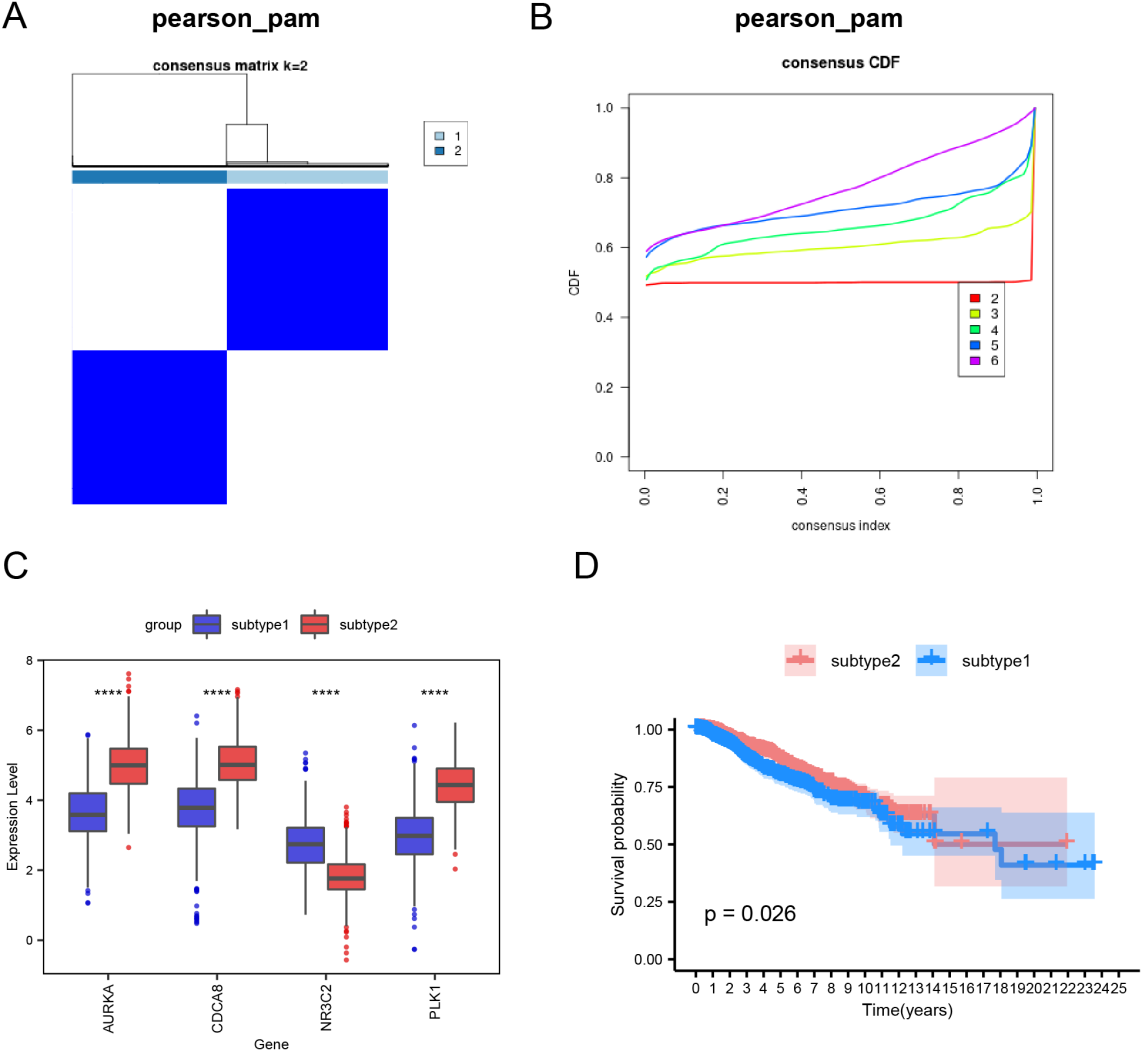
Subtype analysis of BRCA samples using hub genes. **(A)** Clustering heatmap. **(B)** Cumulative distribution function plot. **(C)** Boxplot illustrating hub gene expression across subtype1 and subtype2. **(D)** Survival analysis curve between subtype1 and subtype2. Statistical significance levels are denoted as follows: ****p < 0.0001.

First, based on the four hub genes, we applied consensus clustering to categorize the BRCA samples (n = 1445) into distinct subtypes (**Figures 4A, B**). For clustering, we selected k = 2 and used the pearson_pam algorithm, resulting in two groups: Subtype1 and Subtype2. We then examined the expression of hub genes across the subtypes. Subtype1 showed significantly elevated expression of the critical regulatory gene *NR3C2*, whereas three pivotal cell cycle regulators (*CDCA8*, *AURKA*, and *PLK1)* were markedly overexpressed in Subtype2. These genes showed significant differential expression between the groups (**Figure 4C**). Furthermore, prognostic analysis revealed that there were differences in patient outcomes between the two subtypes, specifically with Subtype2 demonstrating a better prognosis compared to Subtype1 (*p* = 0.026; **Figure 4D**).

### 3.4 Immune infiltration profiling across subtypes

**Figure 5:**
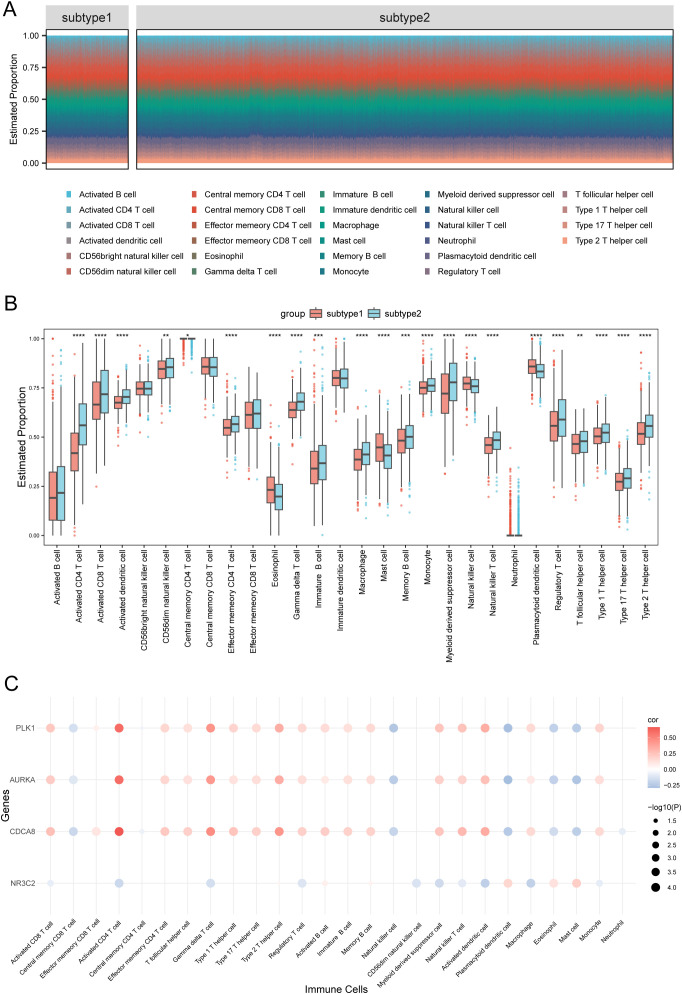
Comparative immune cell infiltration between subtype1 and subtype2. **(A)** Comparative visualization of immune cell distribution in subtype1 versus subtype2 breast carcinoma specimens using segmented columns. **(B)** Boxplot of immune cell proportions between subtypes 1 and 2. **(C)** Bubble plot of the correlation between hub genes and immune cells. Statistical significance levels are denoted as follows: ****p < 0.0001, ***p < 0.001, **p < 0.01, *p < 0.05.

Using ssGSEA, we quantified the immune infiltration of 28 cell types in 1445 BRCA samples (**Figure 5A**, **Supplementary Table S5**). Particularly, we analyzed the differences in the immune infiltration profiles of 28 cell types between Subtype1 and Subtype2. Most immune cells demonstrated significant differences in infiltration between the subtypes, except for activated B cells, CD56bright natural killer cells, central memory CD8+ T cells, effector memory CD8+ T cells, immature dendritic cells, and neutrophils (*p* < 0.05; **Figure 5B**).

Furthermore, we generated a bubble diagram illustrating the interactions between hub genes and immune cell populations (**Figure 5C**). Notably, the four hub genes demonstrated significant associations with 26 of the 28 immune cell types analyzed, indicating their potential regulatory roles in BRCA prognosis through immune modulation.

### 3.5 Inter-subtype GSEA

**Figure 6:**
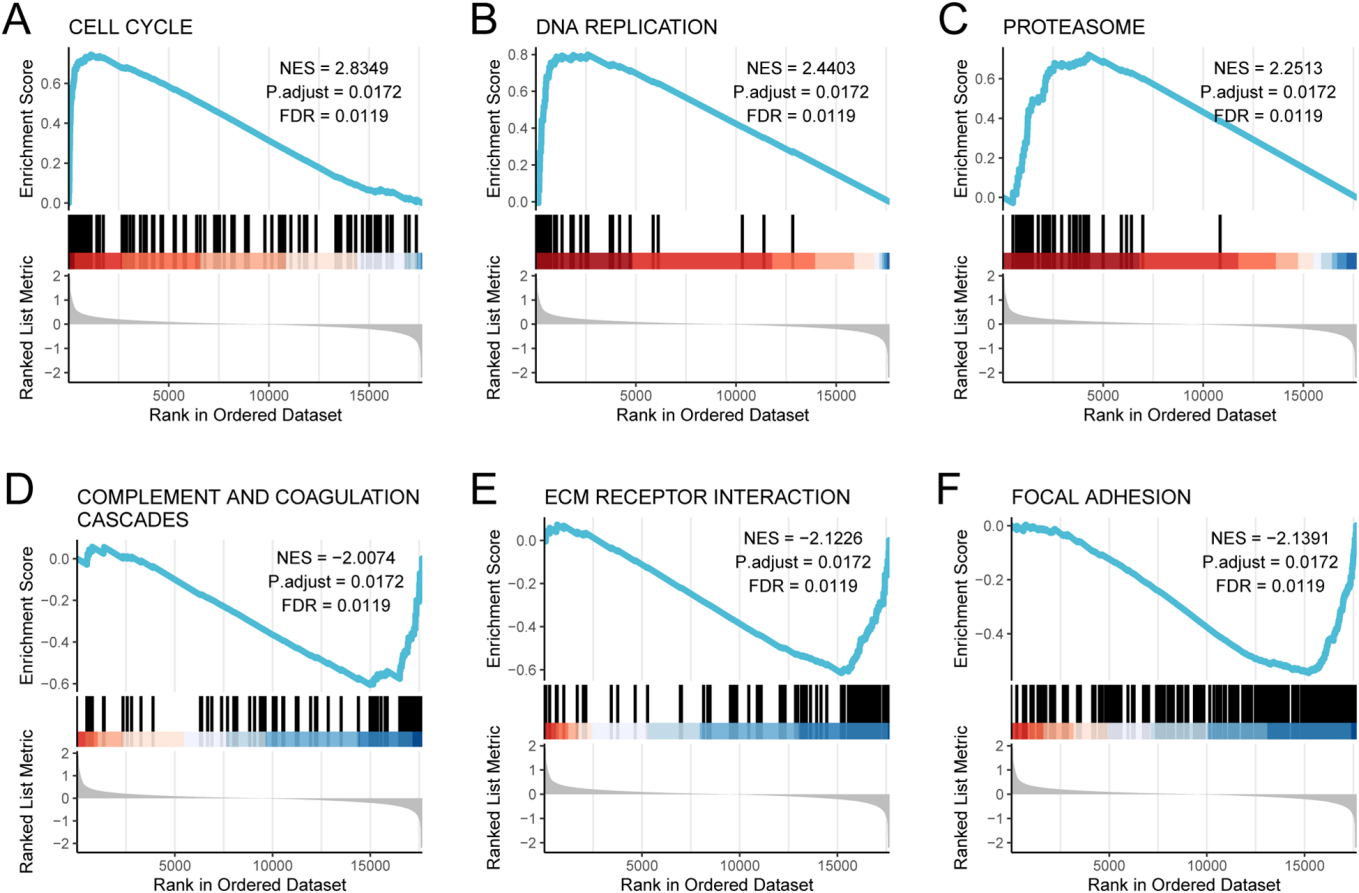
GSEA analysis between subtype1 and subtype2 subtypes. GSEA analysis revealed significant enrichment of **(A)** cell cycle, **(B)** DNA replication, **(C)** proteasome, **(D)** complement and coagulation cascades, **(E)** ECM receptor interaction, and **(F)** focal adhesion.

To investigate the molecular basis of gene expression differences between Subtype1 and Subtype2 and to identify the primary contributors to varying patient risks, we conducted GSEA between these two subtypes. Using pathway data from the MSigDB with a significance threshold of *p* < 0.05, we identified the most significantly enriched pathways based on the Normalized Enrichment Score (NES) ranking (**Supplementary Table S6**). GSEA revealed significant pathway enrichment in Subtype2, with notable activation in the following pathways: cell cycle (NES = 2.8349), DNA replication (NES = 2.4403), and proteasome (NES = 2.2513). All three pathways showed statistical significance (*adj.p* = 0.0172; FDR = 0.0119), as illustrated in **Figures 6A–C**. Conversely, complement and coagulation cascades (NES = −2.0074, *adj.p* = 0.0172, FDR = 0.0119; **Figure 6D**), ECM receptor interaction (NES = −2.1226, *adj.p* = 0.0172, FDR = 0.0119; **Figure 6E**), and focal adhesion (NES = −2.1391, *adj.p* = 0.0172, FDR = 0.0119; **Figure 6F**) were significantly enriched in the Subtype1 than in the Subtype2. Negative NES values indicated relative enrichment in Subtype1 compared to Subtype2.

### 3.6 Regulatory architecture modeling and protein interplay profiling

To explore the interactions between RBPs and mRNAs, we used the StarBase database to identify and download mRNA/RBP pairs associated with the four hub genes. Using target gene interaction data from the dataset, we established an RBP–mRNA regulatory network (**Supplementary Figure S2A**). This comprehensive network architecture comprised 73 nodes (69 RBPs and 4 mRNAs) connected by 156 regulatory edges, demonstrating intricate post-transcriptional regulation patterns.

To investigate the molecular mechanisms mediated by hub genes in BRCA, we established a tripartite RNA interaction network encompassing mRNAs, miRNAs, and lncRNAs. The data revealed only three hub genes (*NR3C2*, *CDCA8*, and *AURKA*) as target mRNAs. The constructed mRNA–miRNA–lncRNA network consisted of 29 nodes, including 9 miRNAs, 3 mRNAs, and 17 lncRNAs, and 116 edges (**Supplementary Figure S2B**).

Using the TRRUST database, we screened TFs interacting with key genes and mapped regulatory networks involving two hub genes (*AURKA* and *PLK1*) and six TFs. These interactions were visualized using Cytoscape software (**Supplementary Figure S2C**).

To investigate the potential functional associations between the identified hub genes, we performed protein–protein interaction (PPI) analysis using the STRING database (https://cn.string-db.org/). Bioinformatics analysis revealed direct PPIs among three cell cycle regulators: *CDCA8*, *AURKA*, and *PLK1* (**Supplementary Figure S2D**).

In summary, our network analyses revealed extensive post-transcriptional interactions focused on the hub genes, encompassing RBP regulation with 69 proteins, a ceRNA network of 29 nodes, transcriptional modulation by 6 transcription factors, and direct protein interactions among CDCA8, AURKA and PLK1, highlighting their central roles in BRCA regulatory pathways.

### 3.7 Analysis of TMB, TIDE, immune checkpoints, and drug sensitivity between subtypes

**Figure 7:**
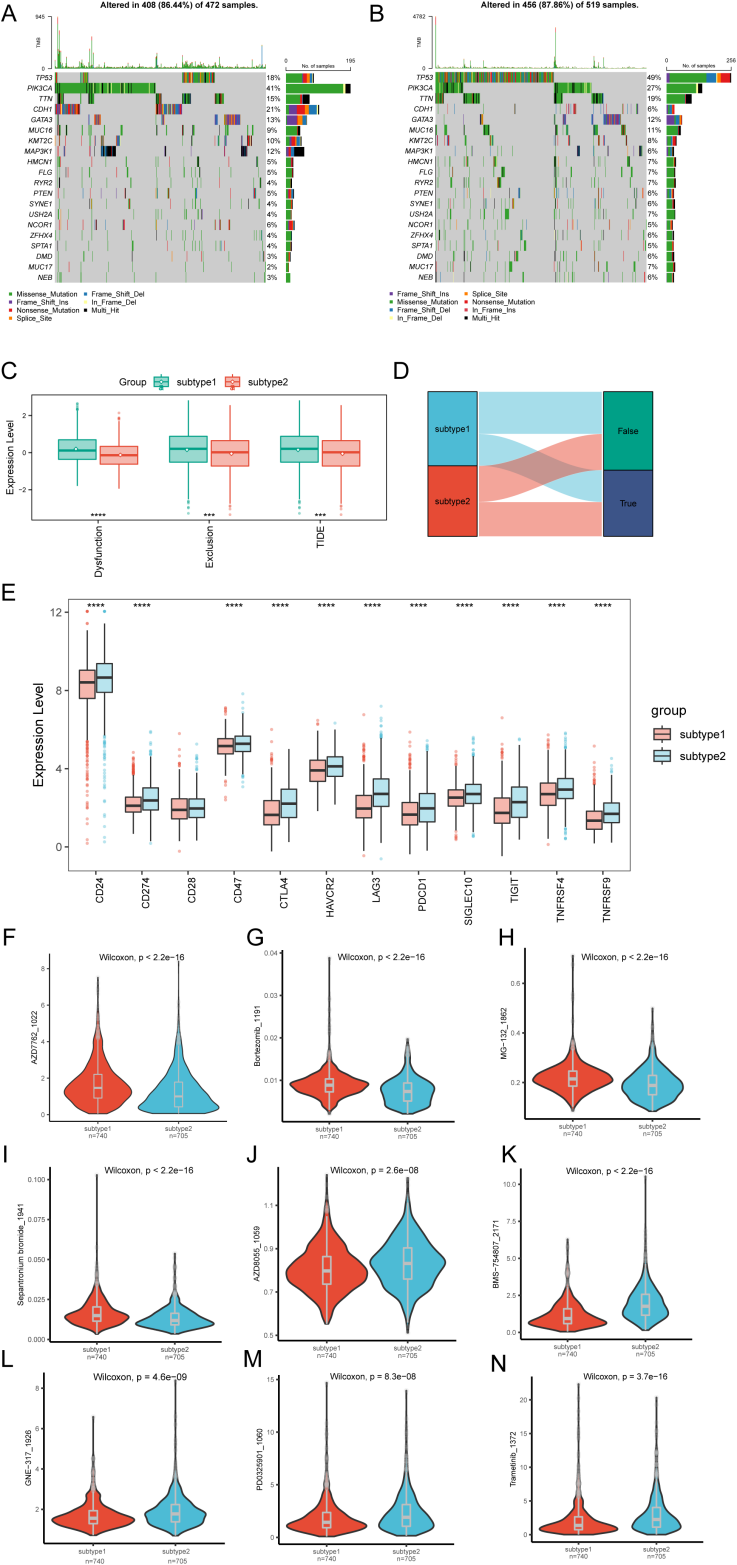
Differential analysis of TMB, TIDE, immune checkpoints, and drug response in molecular subtypes. **(A)** Top-ranked 20 mutational hotspots identified in subtype1 population. **(B)** Leading 20 genes demonstrating predominant mutational rates within subtype2. **(C)** Boxplot of TIDE analysis between subtype1 and subtype2 subtypes. **(D)** Sankey diagram of TIDE analysis between subtype1 and subtype2 subtypes. **(E)** Boxplot showing the expression of immune checkpoints between subtype1 and subtype2 subtypes. Differential drug sensitivity between subtype1 and subtype2 subtypes for **(F)** AZD7762_1022, **(G)** AZD8055_1059, **(H)** BMS-754807_2171, **(I)** Bortezomib_1191, **(J)** GNE-317_1926, **(K)** MG-132_1862, **(L)** PD0325901_1060, **(M)** Sepantronium bromide_1941, **(N)** Trametinib_1372. Statistical significance levels are denoted as follows: ****p < 0.0001, ***p < 0.001.

To evaluate specific gene mutations in BRCA, we conducted a TMB analysis between Subtype1 and Subtype2, highlighting the 20 most FMGs. Within these groups, PIK3CA exhibited the highest mutation rates in Subtype1, whereas TP53 had the greatest mutational prevalence in Subtype2 (**Figures 7A, B**).The TIDE analysis results comparing the two molecular subtypes are presented in **Figures 7C, D**. Subtype2 exhibited significantly lower TIDE scores (Tumor Immune Dysfunction and Exclusion scores) and exclusion scores compared to Subtype1. Conversely, Subtype1 demonstrated elevated dysfunction scores compared to Subtype2. This differential pattern implies that Subtype1 may possess greater potential for immune evasion mechanisms, as evidenced by its distinct TIDE profile characteristics. Comparative analysis of immune checkpoint expression revealed significant differences between Subtype1 and Subtype2. All evaluated genes, except *CD28*, demonstrated statistically significant differential expression across the groups (**Figure 7E**), suggesting distinct immunotherapy response potentials between the molecular subtypes.

We further assessed the predictive accuracy of molecular subtypes for chemotherapy response in patients with BRCA. The clinical efficacies of several chemotherapeutic drugs in BRCA treatment were investigated (**Supplementary Table S7**). The findings suggest that patients with Subtype1 might be more responsive to chemotherapy with AZD7762_1022 (**Figure 7F**), Bortezomib_1191 (**Figure 7G**), MG-132_1862 (**Figure 7H**), and Sepantronium bromide_1941 (**Figure 7I**). Conversely, patients with Subtype2 might respond more sensitively to chemotherapy with AZD8055_1059 (**Figure 7J**), BMS-754807_2171 (**Figure 7K**), GNE-317_1926 (**Figure 7L**), PD0325901_1060 (**Figure 7M**), and Trametinib_ 1372 (**Figure 7N**). These results suggest the potential regulatory effects of chemotherapeutic drugs on SUMOylation.

### 3.8 Drug-gene interaction analysis

A search of the DGIdb for drugs targeting the four BRCA-related hub genes revealed three significant interactions: *AURKA* with the Aurora A kinase inhibitor MK5108, *NR3C2* with Finerenone, and *PLK1* with MK-1496, which exhibited the strongest binding affinities (**Table 1**). This pharmacological analysis identified specific drug-gene pairs with maximal interaction scores within the database.

**Table 1:** Drug prediction.

Gene	Drug	Interaction score
AURKA	Aurora A kinase inhibitor MK5108	1.8
NR3C2	Finerenone	4.12
PLK1	MK-1496	0.58

### 3.9 Pan-cancer molecular signatures with prognostic implications

**Figure 8:**
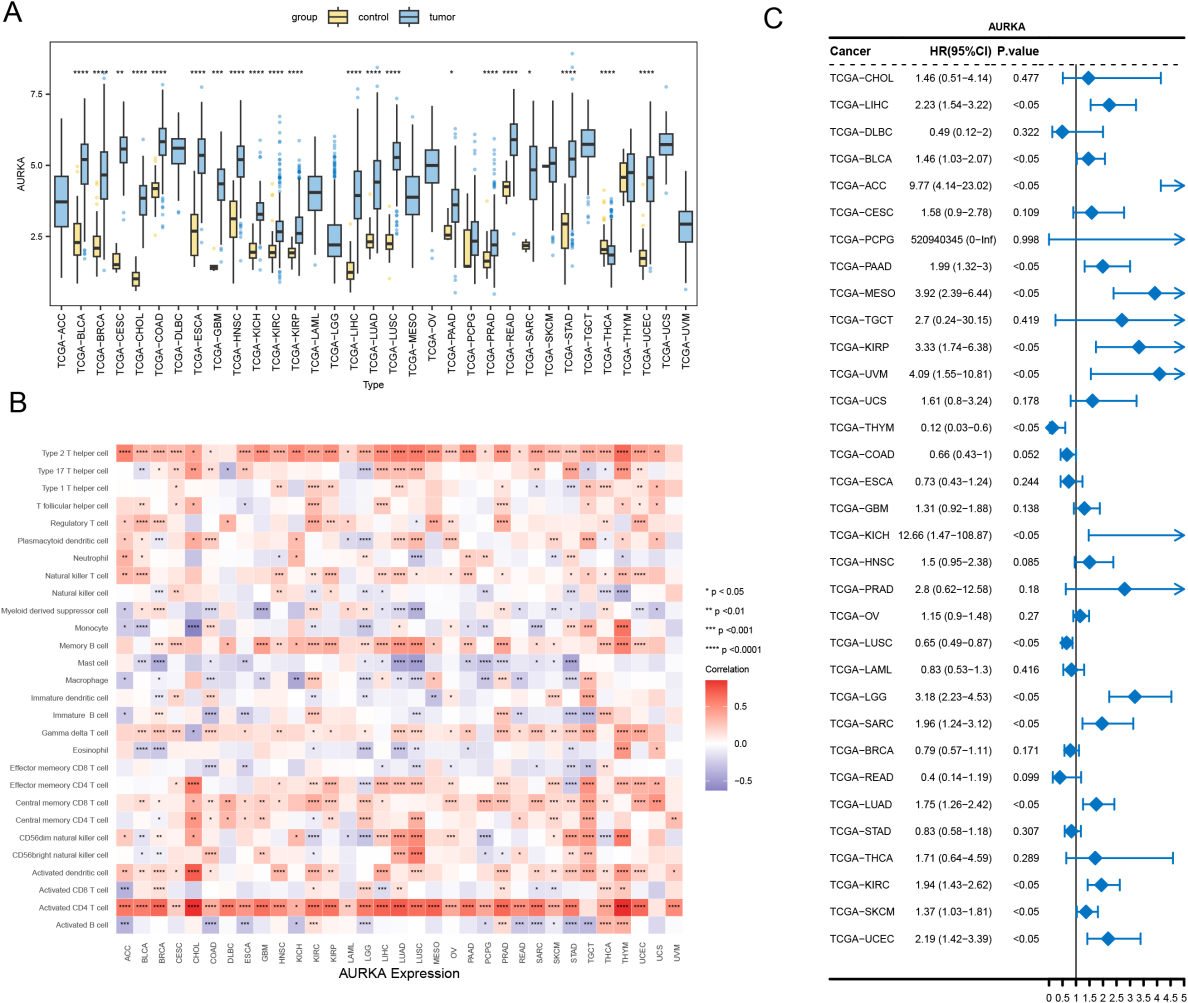
Pan-cancer biomarker prognostication. **(A)** Boxplot showing the expression of *AURKA* across multiple tumor types. **(B)** Thermal map depicting *AURKA*-immunocyte associations across cancers. **(C)** Pan-cancer forest graph illustrating univariate Cox analysis of *AURKA* expression. Statistical significance levels are denoted as follows: ****p < 0.0001, ***p < 0.001, **p < 0.01, *p < 0.05.

The expression profiles of the four hub genes across various cancers are illustrated using boxplots. Focusing on the prognostic gene *AURKA* as an example (results for other prognostic genes are shown in **Supplementary Figures S3–S5**), *AURKA* expression differed significantly between the two groups. *AURKA* was significantly upregulated in multiple malignancies, including bladder urothelial carcinoma (BLCA), breast invasive carcinoma (BRCA), cervical squamous cell carcinoma/endocervical adenocarcinoma (CESC), cholangiocarcinoma (CHOL), and gastrointestinal cancers (COAD, ESCA, STAD, and READ), compared to normal tissues, along with notable increases in glioblastoma (GBM), head–neck squamous carcinoma (HNSC), renal neoplasms (KICH, KIRC, and KIRP), hepatic carcinoma (LIHC), pulmonary malignancies (LUAD and LUSC), pancreatic adenocarcinoma (PAAD), prostate adenocarcinoma (PRAD), sarcoma (SARC), and endometrial carcinoma (UCEC). Conversely, thyroid carcinoma (THCA) demonstrated significant downregulation (**Figure 8A**). Subsequent pan-cancer analysis revealed correlations between *AURKA* expression patterns and 28 immune cell subtypes across 33 malignancies (**Figure 8B**). Prognostic evaluation using Cox regression modeling revealed significant associations between *AURKA* expression levels and overall survival in 16 cancer types. Elevated expression correlated with poorer outcomes in LIHC, BLCA, adrenocortical carcinoma (ACC), PAAD, mesothelioma (MESO), KIRP, uveal melanoma (UVM), KICH, lower-grade glioma (LGG), SARC, LUAD, KIRC, cutaneous melanoma (SKCM), and UCEC, while demonstrating protective effects in LUSC and thymoma (THYM) (**Figure 8C**).

### 3.10 Dimensionality reduction in single-cell data

**Figure 9:**
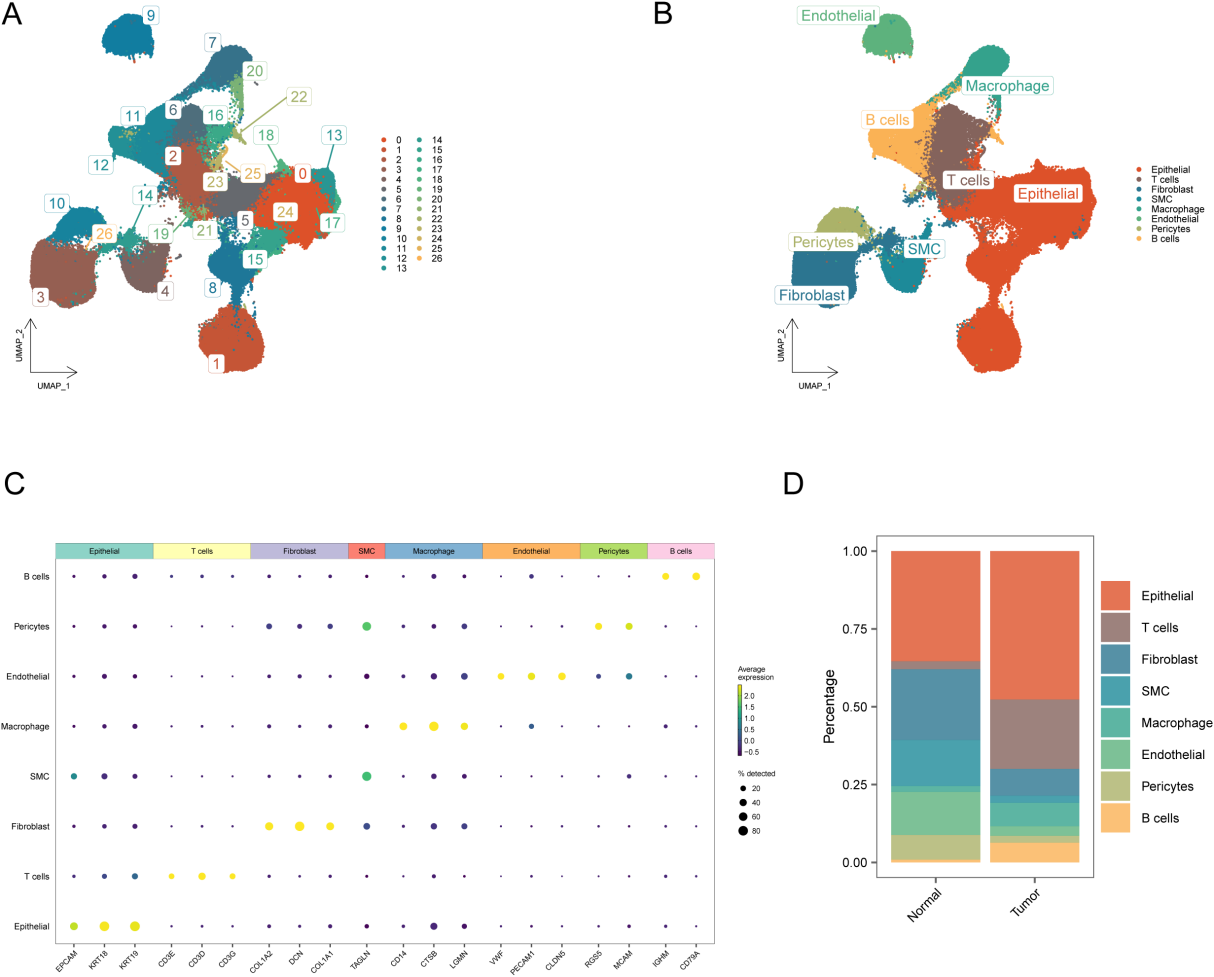
Annotation and visualization of the cellular microenvironment in breast cancer. **(A)** UMAP visualization delineating cellular subset segregation between tumor and normal cohorts; **(B)** UMAP visualization of cellular cluster annotations between malignant and benign specimens; **(C)** Transcriptomic signatures characterizing eight distinct cellular lineages; **(D)** Cumulative histogram illustrating cellular composition variations in neoplastic versus healthy cohorts.

To investigate cellular heterogeneity in BRCA, we analyzed the single-cell sequencing dataset GSE161529. After rigorous quality control and pre-processing steps, 357,292 high-quality cells were identified through transcriptomic profiling. Cells were partitioned into 27 distinct subpopulations using unsupervised clustering analysis (**Figure 9A**). Cellular identities were subsequently determined by analyzing the transcriptional profiles of individual clusters, supplemented with the known lineage markers (**Figures 9B, C**, **Supplementary Table S8**). **Figure 9B** delineates eight principal cellular populations: T and B lymphocytes, macrophages, endothelial and epithelial lineages, fibroblasts, pericytes, and smooth muscle cells.

Finally, comparative analysis revealed distinct variations in cellular composition between the BRCA and control groups, with detailed population ratios depicted in **Figure 9D**. Notably, the proportion of fibroblasts showed significant changes before and after the onset of BRCA.

### 3.11 Screening of key cell types

**Figure 10:**
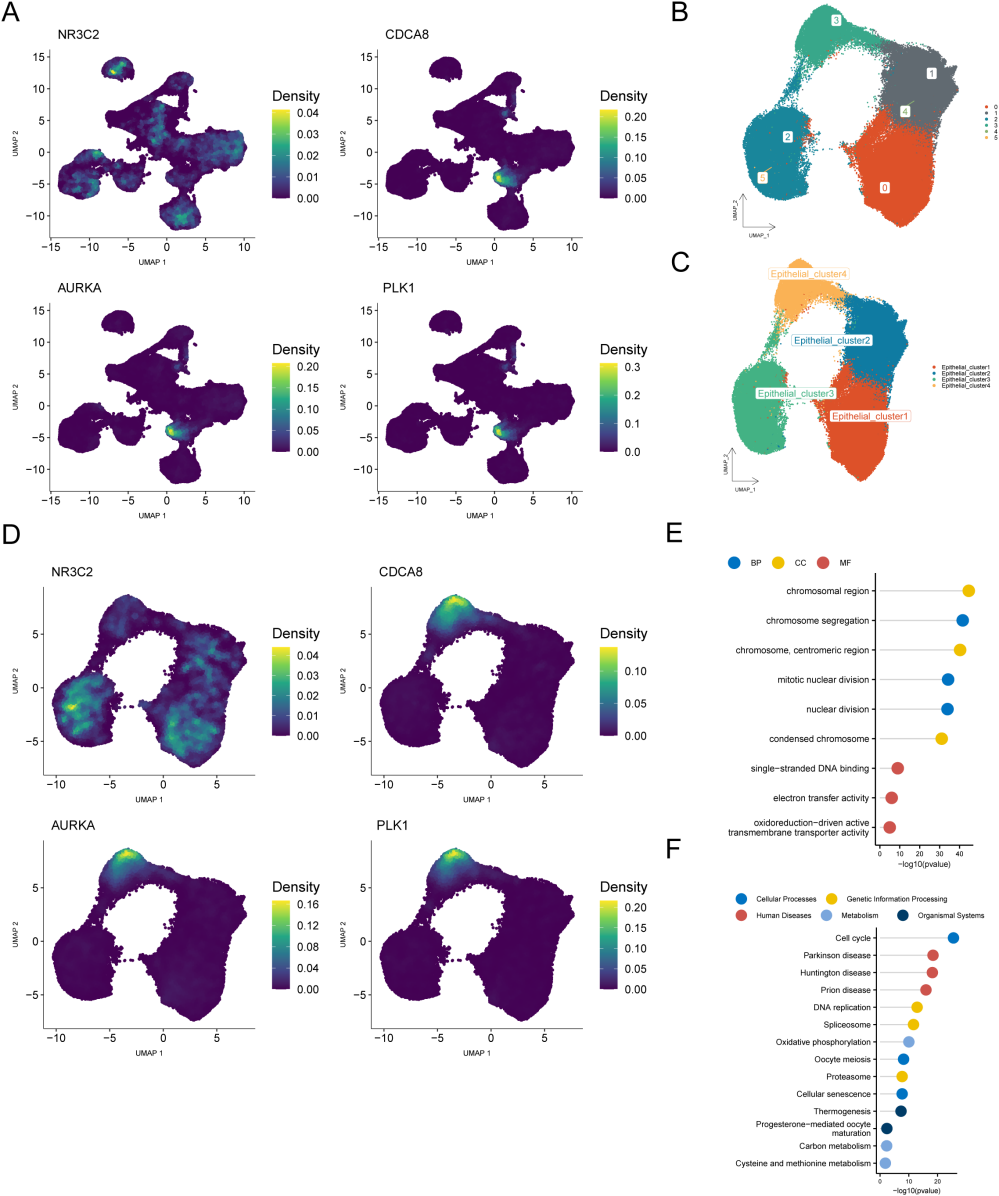
Identification and exploration of key cell populations based on hub genes. **(A)** UMAP plot showing the distribution of hub genes across cell subpopulations. **(B, C)** Re-clustered and annotated UMAP plot of Epithelial cells. **(D)** UMAP plot displaying the distribution of hub genes across four Epithelial cell subclusters. **(E)** Lollipop plot of GO enrichment analysis for differential genes between Epithelial_cluster4 and the other three Epithelial cell subclusters. **(F)** Lollipop plot of KEGG enrichment analysis for differential genes between Epithelial_cluster4 and the other three Epithelial cell subclusters.

To identify the critical cellular subtypes involved in BRCA pathogenesis, we spatially mapped the four central regulators within the single-cell transcriptional profiles. The findings revealed that most of these hub genes were clustered within the epithelial cell population, identifying epithelial cells as the key cell group (**Figure 10A**). We subsequently re-clustered the epithelial cells (resolution = 0.2) and identified six subclusters, which were labeled as four cell types: Epithelial_cluster1, Epithelial_cluster2, Epithelial_cluster3, and Epithelial_cluster4 (**Figures 10B, C**). Mapping the four hub genes onto these four cell subclusters indicated that the majority were clustered within the Epithelial_cluster4 (**Figure 10D**). Therefore, we analyzed the differential genes between the key cell subcluster Epithelial_cluster4 and the other three subclusters, identifying 830 differential genes (logFC = 0.25, *p* < 0.05; **Supplementary Table S9**). Enrichment analyses were performed for GO categories (**Supplementary Table S10**) and KEGG pathways based on the DEGs (**Supplementary Table S11**). Functional annotation revealed significant enrichment in key biological processes, such as chromosome segregation and mitotic nuclear division (BP), with predominant cellular localization observed in chromosomal domains, including centromeric regions and condensed chromosomes (CC). Molecular characterization revealed enhanced functionality in single-stranded DNA binding and oxidoreductase-driven transmembrane transport activities (MF) (**Figure 10E**).

KEGG pathway analysis revealed significantly enriched pathways in various categories: (1) cellular processes: cell cycle, oocyte meiosis, and cellular senescence; (2) human diseases: Parkinson’s disease, Huntington’s disease, and prion disease; (3) genetic information processing: DNA replication, spliceosome, and proteasome; (4) metabolism: oxidative phosphorylation, carbon metabolism, and cysteine and methionine metabolism; and (5) organismal systems: thermogenesis and progesterone-mediated oocyte maturation (**Figure 10F**).

### 3.12 Analysis of TFs in cell subclusters

**Figure 11:**
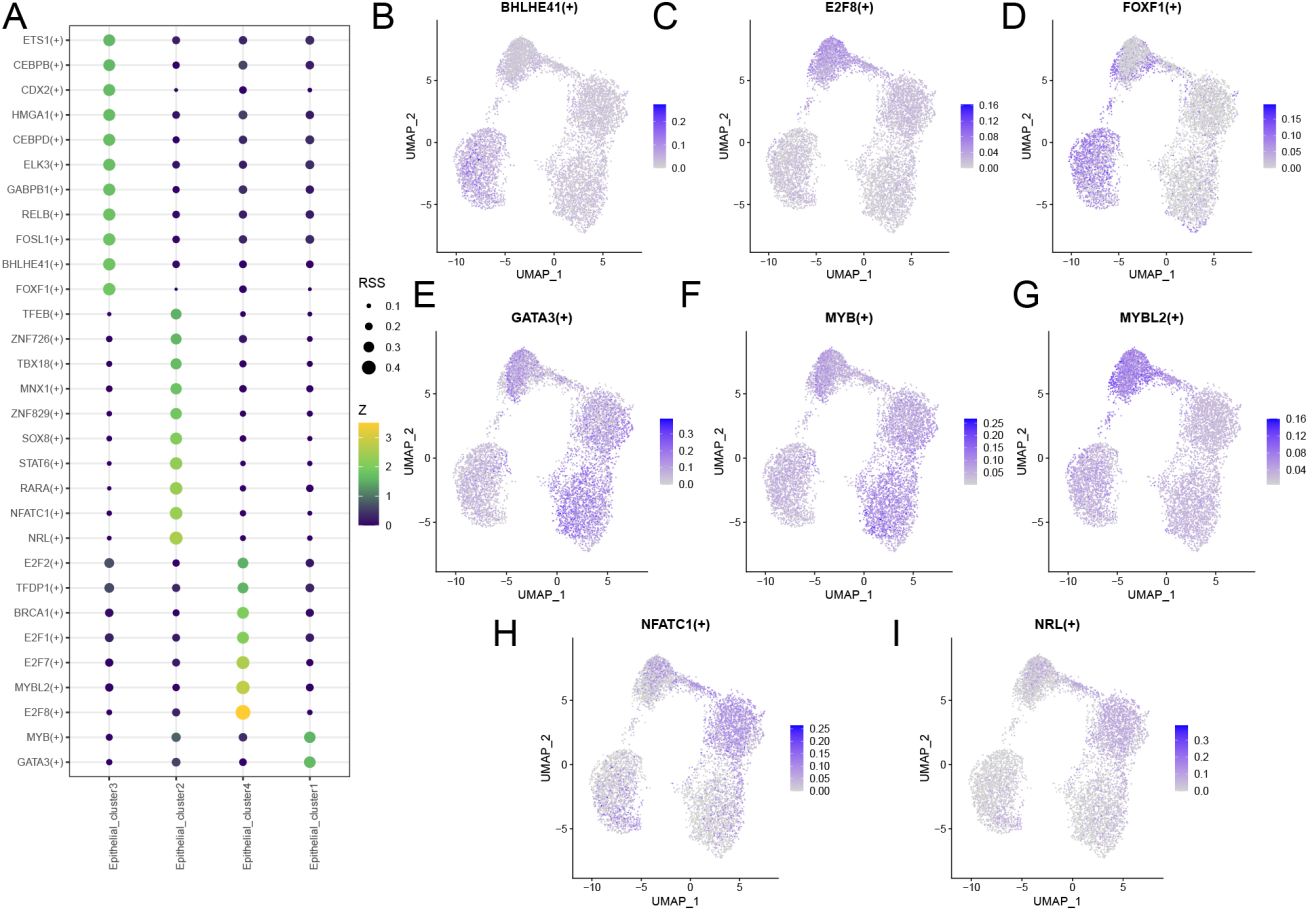
Transcription factor analysis of epithelial cells. **(A)** Bubble plot showing specific transcription factors in cell types. The bubble size represents the RSS (Regulon Specificity Score) value of the TF in a specific cell type. Elevated RSS values correlate with both increased bubble size and enhanced transcription factor specificity within the respective cell type. The color represents the Z-score, where a higher score indicates that the TF expression level in the specific cell type is significantly higher than in other cell types. **(B-I)** UMAP plots displaying the enrichment of eight transcription factors across four Epithelial cell subclusters. TFs, transcription factors.

To investigate the transcriptional regulation of BRCA pathogenesis, we first analyzed the specific TFs in the four cell subclusters (**Figure 11A**). A total of 30 TFs with cell-specific expression were identified, among which *E2F8*, *MYBL2*, *GATA3*, *MYB*, *NRL*, *NFATC1*, *FOXF1*, and *BHLHE41* exhibited strong specificity for their respective cell types. Analysis of the enrichment levels of these eight TFs in the four cell subclusters revealed that *GATA3* and *MYB* were enriched in Epithelial_cluster1 cells; *NFATC1* and *NRL* were enriched in Epithelial_cluster2 cells; *BHLHE41* and *FOXF1* were enriched in Epithelial_cluster3 cells; and *E2F8*, *GATA3*, *MYB*, and *MYBL2* were enriched in Epithelial_cluster4 cells (**Figures 11B–I**). These findings suggest that these TFs may play pivotal roles in breast carcinogenesis.

### 3.13 Prediction of drugs targeting key TFs

We analyzed eight key TFs using the DGIdb (https://www.dgidb.org/), sorting the results in descending order based on interaction score and selecting the small-molecule drugs with the highest scores as the final predictions. Ultimately, only five TFs, namely *NFATC1*, *GATA3*, *FOXF1*, *E2F8*, and *MYB*, successfully predicted small-molecule drugs: Mycophenolate (for NFATC1), Merimepodib (for *GATA3*), Bevacizumab (for *FOXF1*), Edifoligide (for *E2F8*), Edifoligide Sodium (for *E2F8*), and Retinoic Acid Agent (for *MYB*) (**Table 2**).

**Table 2:** Small molecule drug screening.

Transcription factor	Drug	Interaction score
NFATC1	Mycophenolate	1.19
GATA3	Merimepodib	1.37
FOXF1	Bevacizumab	1.31
E2F8	Edifoligide, Edifoligide Sodium	3.26
MYB	Retinoic Acid Agent	0.56

## 4 Discussion

BRCA is the most prevalent malignancy globally, with approximately 2.3 million new cases and 684,996 deaths annually (2). Despite advancements in chemotherapy and targeted therapies, many patients still experience drug resistance and metastasis (3), highlighting the limitations of current treatment approaches. Recent research has emphasized the regulatory functions of SUMOylation in BRCA, particularly in maintaining genome stability and modulating TFs (42). However, the specific roles of SUMOylation-associated hub genes in tumorigenesis and heterogeneity remain poorly understood. Significant research gaps exist in mapping the dynamic SUMOylation networks across BRCA subtypes and developing subtype-specific therapeutic strategies targeting this pathway.

We systematically identified *CDCA8*, *AURKA* and *PLK1* as hub regulators in BRCA-specific cell cycle networks, with integrated bulk and single-cell RNA sequencing data confirming their prognostic significance (**Figures 3G, I**, **10A**). This integration of established mechanisms and novel discoveries enhances insights into potential therapeutic targets, while validating methodological robustness.

Integrated transcriptomic and single-cell profiling identified a pivotal regulatory network centered on *NR3C2*, *CDCA8*, *AURKA*, and *PLK1* in breast carcinogenesis. *NR3C2* displayed complex, context-dependent functionality: although it was significantly downregulated in tumors overall—suggesting a potential tumor-suppressive role (**Figures 3I, J**) (43)—its elevated expression in Subtype1 correlated with poorer outcomes (**Figures 4C, D**). In contrast, *CDCA8*, *AURKA*, and *PLK1* were consistently overexpressed (44) and exhibited direct protein interactions (**Supplementary Figure S2D**). Intriguingly, the collective high expression of these three regulators defined Subtype2, which was associated with a favorable prognosis despite their established pro-oncogenic functions (**Figures 3I, J**, **4C, D**) (45). This functional paradox underscores their context-dependent activity (46–48), further illustrated by Subtype2’s enhanced sensitivity to targeted therapies such as Trametinib (**Figure 7N**) (49). These molecular subtypes also displayed distinct immune landscapes (**Figures 5A, B**) and mutational spectra (**Figures 7A, B**). Single-cell resolution further pinpointed hub gene enrichment within specific epithelial subpopulations (**Figures 10A–D**). Collectively, our findings establish a novel molecular taxonomy grounded in SUMOylation-associated networks, linking transcriptional patterns with therapeutic vulnerabilities to advance precision oncology.

In this study, we utilized GO and KEGG enrichment analyses to identify the key pathways associated with hub genes in BRCA development. The results highlighted the cell cycle regulation, DNA replication, and senescence mechanisms drive tumor progression (**Supplementary Table S4**; **Figures 2I, J**). As a fundamental biological process controlling cellular division, the pathological disruption of cell cycle control emerges as a critical oncogenic feature in BRCA. The observed enrichment of cell cycle-related genes suggests that these regulatory networks may significantly influence abnormal proliferation patterns in cancerous growths (**Figures 2I, J**).

The DNA replication pathway maintains genomic stability, and its dysregulation may drive cancer-associated genomic instability (**Figure 6B**; **Supplementary Table S6**). The hub genes identified in this study potentially promote BRCA progression by abnormally regulating these pathways. Although cellular senescence typically suppresses tumor formation, the accumulation of senescent cells paradoxically stimulates cancer growth through the persistent secretion of inflammatory factors that establish pro-tumorigenic microenvironments via paracrine signaling (**Figure 2J**; **Supplementary Table S4**). These pathway discoveries deepen our understanding of BRCA biology and reveal potential therapeutic targets for it. Intervention strategies focusing on these pathways could address current treatment limitations and improve the clinical outcomes. This study underscores the critical involvement of these pathways in breast carcinogenesis and their translational potential for developing targeted therapies and contributing to the understanding of molecular features of breast cancer.

Our analysis revealed subtype-specific immune signatures (Subtype1 vs. Subtype2), consistent with previous findings on the bidirectional role of the TME in tumor progression (50) (**Figures 5A, B**). Increased infiltration of activated CD8+ T cells and macrophages correlated with improved survival outcomes, highlighting the prognostic significance of anti-tumor immunity (51) (**Figures 4D**, **5B**). Critically, we identified hub genes that serve as dual biomarkers, predicting both prognosis and immunotherapy response—a functional extension of existing studies (**Figures 4D**, **7C**). Our study significantly expands the understanding of these hub genes by revealing their pivotal role in reprogramming the tumor immune microenvironment. These findings advance precision therapeutics by integrating subtype-specific immune profiles with gene networks, facilitating strategies to boost treatment efficacy through microenvironmental targeting and addressing unmet clinical needs in BRCA management (52).

In the current investigation, we successfully identified 357,292 cells using scRNA-seq and categorized them into 27 distinct cell clusters (**Figure 9A**), primarily comprising T cells, B cells, and macrophages (**Figures 9B, C**). This comprehensive cellular profiling highlights the significant heterogeneity within the BRCA microenvironment, which is crucial for understanding tumor biology and therapeutic responses. The identification of these cell types and their respective clusters provides a foundational dataset for future investigations into the molecular pathways underlying tumor evolution and therapeutic resistance in mammary carcinoma. The role of T cells in mediating anti-tumor immunity and the involvement of macrophages in promoting tumor growth and immune evasion are well documented (53, 54). Furthermore, the heterogeneity within neoplastic microenvironments, characterized by interactions among various immune cells (**Figure 9D**), highlights the necessity for targeted therapeutic strategies that can modulate these interactions for improved therapeutic outcomes.

Although our findings provide crucial insights into the cellular heterogeneity within BRCA tumors, several limitations should be acknowledged. For instance, reliance on scRNA-seq data may not fully capture the spatial organization and functional states of cells within the TME. Moreover, the analysis does not account for temporal changes in cell populations during disease progression or treatment. Future studies that integrate spatial transcriptomics and longitudinal sampling could greatly enhance our understanding of TME dynamics.

Our results reveal the intricate cellular landscape of BRCA, emphasizing the importance of integrated analysis of bulk RNA sequencing data in elucidating the mechanisms underlying tumor heterogeneity and guiding the development of personalized therapeutic strategies. A deeper understanding of the immune microenvironment may open avenues for novel treatments aimed at improving clinical outcomes and overcoming therapy resistance (55). Based on transcriptomic correlation analysis, the conclusions drawn from this study require further validation through proteomics and functional experiments to elucidate the underlying mechanisms.

## Data Availability

The original contributions presented in the study are publicly available. This data can be found here: https://github.com/hewenxing2012/SUMOylation-BRCA-SingleCell-Bioinformatics.
