# Supplementary material for: Significance of SUMOylation in breast cancer progression: a comprehensive investigation using single-cell analysis and bioinformatics
Source: Front Immunol. 2025 Nov 20;16:1675874. doi: 10.3389/fimmu.2025.1675874 (PMC12676025; doi:10.3389/fimmu.2025.1675874)
Supplement: Supplementary file 1 [file DataSheet1.docx]

**Supplementary files**

**Figure S:**

Fig. S1 PCA results comprising two datasets: GSE20685 (red circles) and TCGA-BRCA (blue triangles). The horizontal axis represents the first principal component (Dim1), accounting for 8.7% of variance, while the vertical axis displays the second component (Dim2), explaining 7.1% variance. Ellipse boundaries in the scatter plot delineate data distribution ranges, demonstrating distinct clustering patterns and spatial separation between the two groups.

Fig. S2. Construction of Hub Gene Regulatory Networks. (A) RBP-mRNA regulatory axis in core genes. Blue boxes represent RBPs (RNA-binding proteins), and yellow circles represent mRNAs. (B) Core gene interaction network involving lncRNA-miRNA-mRNA crosstalk (color-coded: red-lncRNAs, blue-miRNAs, yellow-mRNAs). (C) Core mRNA-TF interconnectome (mRNA: yellow; TF: blue). (D) Hub gene interaction interconnectivity map.

Fig. S3 Multi-tumor predictive marker assessment focusing on *CDCA8*.

(A) A box-and-whisker plot comparing *CDCA8* expression across multiple cancer types, stratified into control (yellow) and tumor (blue) groups. Statistical significance levels are denoted as follows: *****p* < 0.0001, ****p* < 0.001, ***p* < 0.01, **p* < 0.05.

(B) A heatmap illustrating correlations between *CDCA8* expression and various immune cell subtypes, where the color bar indicates both magnitude and direction of correlation coefficients (deep red: positive association; navy blue: negative association).

(C) Prognostic values through hazard ratios (HR) with corresponding 95% confidence intervals (CI) and *p*-values across different malignancies. The horizontal axis represents HR values, with leftward arrows indicating the numerical range of HR estimates. This panel provides quantitative assessment of *CDCA8*'s clinical relevance in oncological outcomes.

Fig. S4 Multi-tumor predictive marker assessment focusing on *NR3C2*.

(A) Boxplots of *NR3C2* expression across multiple cancer types, stratified into control (yellow) and tumor (blue) groups.Statistical significance levels are denoted as follows: *****p* < 0.0001, ****p* < 0.001, ***p* < 0.01, **p* < 0.05.

(B) A heatmap illustrating correlations between *NR3C2* expression and various immune cell subtypes, accompanied by a color scale depicting the strength and direction of correlation coefficients (deep red: positive, navy blue: negative).

(C) Hazard ratios (HR) with corresponding 95% confidence intervals (CI) and *p*-values for different cancers, providing a quantitative analysis of *NR3C2* expression's prognostic significance across malignancies. The horizontal axis represents HR, with leftward arrows indicating the range.

Fig. S5 Multi-tumor predictive marker assessment focusing on *PLK1*.

(A) Comparative boxplots of *PLK1* expression across multiple cancer types, stratified into control (yellow) and tumor (blue) groups.Statistical significance levels are denoted as follows: *****p* < 0.0001, ****p* < 0.001, ***p* < 0.01, **p* < 0.05.

(B) A heatmap illustrating correlation patterns between *PLK1* expression and various immune cell subtypes, with the color gradient denoting both magnitude and direction of correlation coefficients (deep red for positive associations, navy blue for inverse relationships).

(C) Forest plot visualization of prognostic parameters, enumerating hazard ratios (HR) with corresponding 95% confidence intervals (CI) and *p*-values across distinct malignancies. The horizontal axis scales HR values, while leftward arrows demarcate the range of effect estimates, offering a quantitative assessment of *PLK1* expression's prognostic significance in oncological outcomes.

**Table S**

Table S1: SUMOylation-related genes.

Table S2: DEGs in BRCA vs. control.

Table S3: GO enrichment analysis results.

Table S4: KEGG pathway enrichment analysis results.

Table S5: Immune cell infiltration profiles.

Table S6: GSEA results for subtype comparison.

Table S7: Chemotherapy sensitivity predictions.

Table S8: Cellular cluster annotations (scRNA-seq).

Table S9: Differential genes in Epithelial_cluster4.

Table S10: GO analysis for Epithelial_cluster4 genes.

Table S11: KEGG analysis for Epithelial_cluster4 genes.
