# Supplementary figures and images for: Significance of SUMOylation in breast cancer progression: a comprehensive investigation using single-cell analysis and bioinformatics
Source: Front Immunol. 2025 Nov 20;16:1675874. doi: 10.3389/fimmu.2025.1675874 (PMC12676025; doi:10.3389/fimmu.2025.1675874)

Individuals – PCA

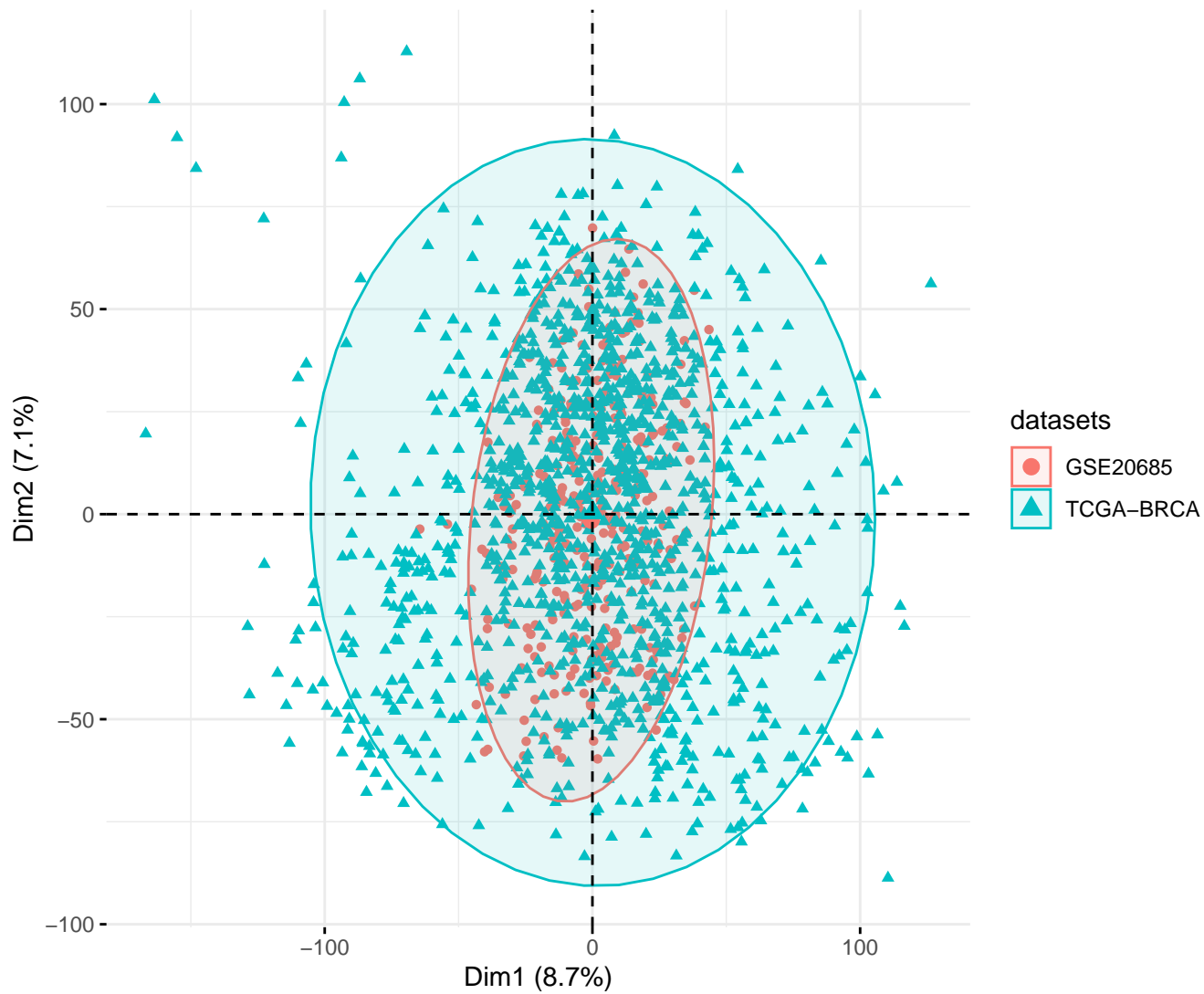

Supplement: Supplementary file 2 [file Image1.pdf]

A

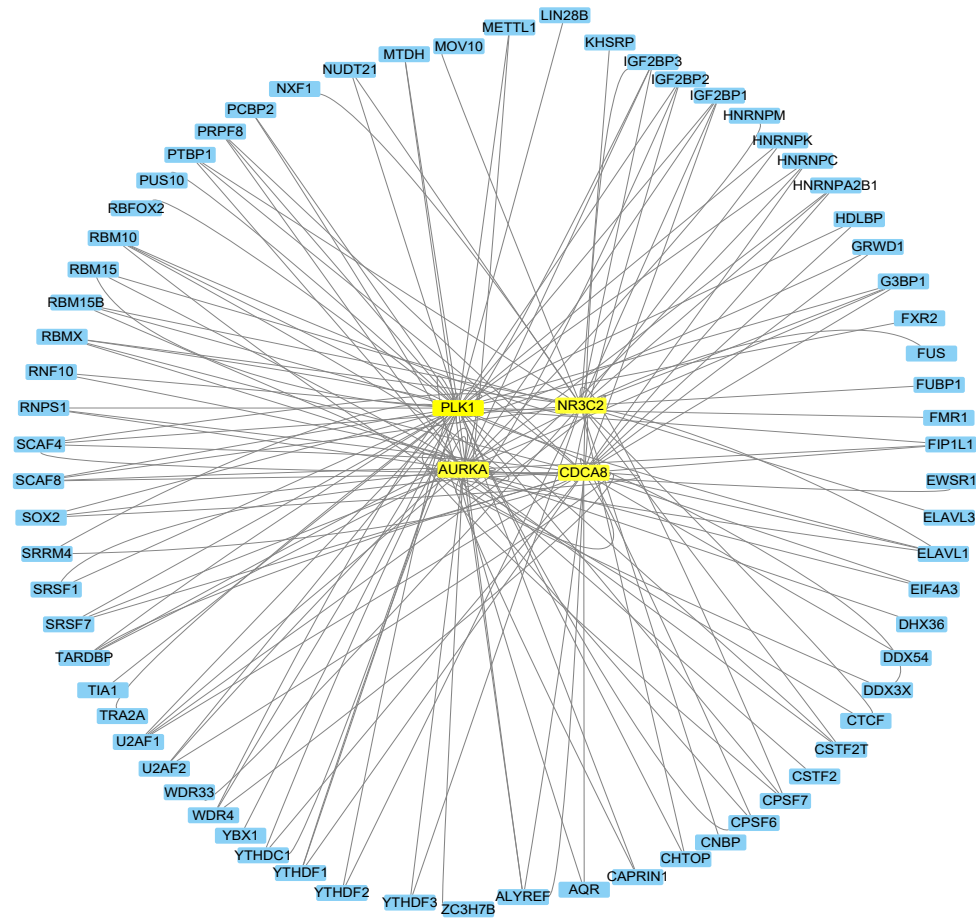

B

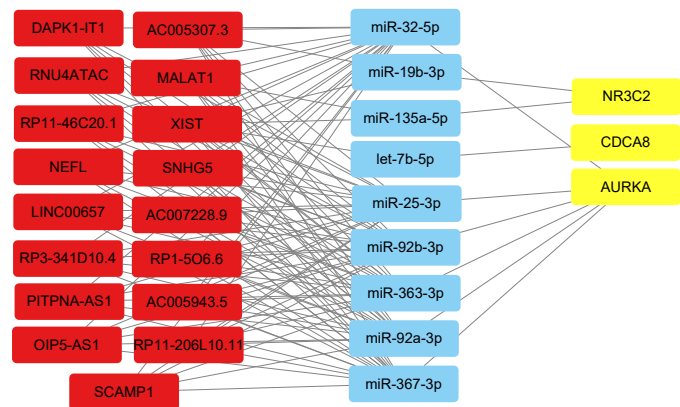

C

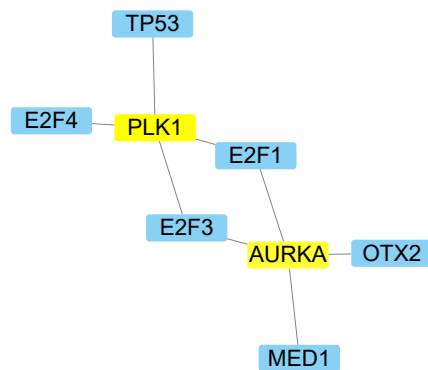

D

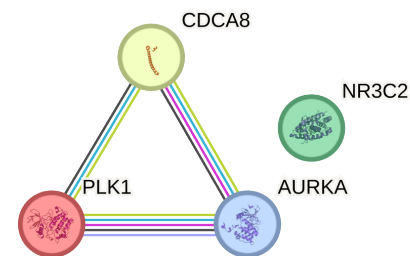

Supplement: Supplementary file 3 [file Image2.pdf]

A

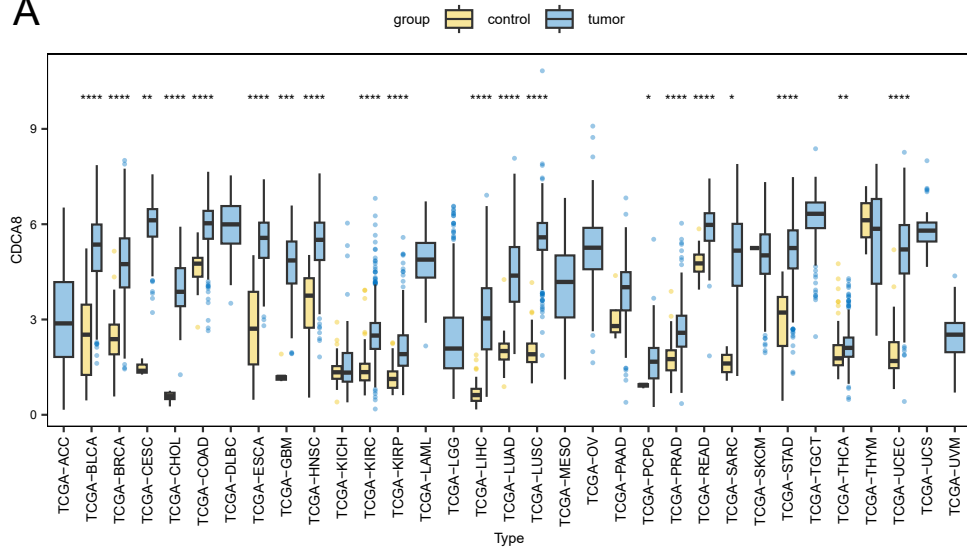

B

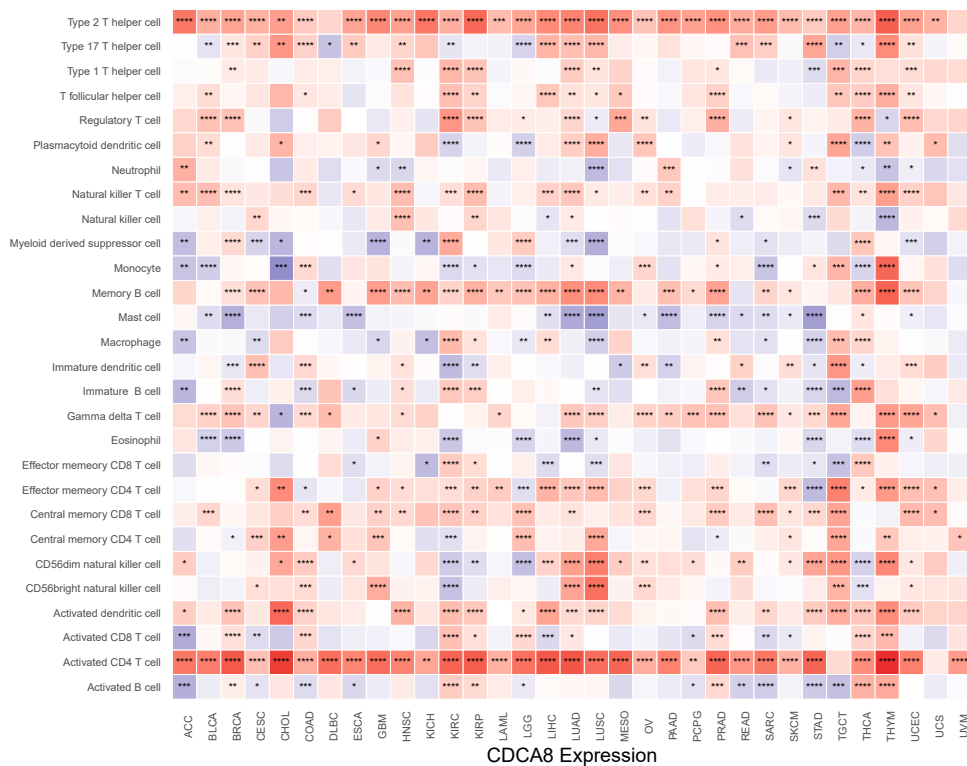

C

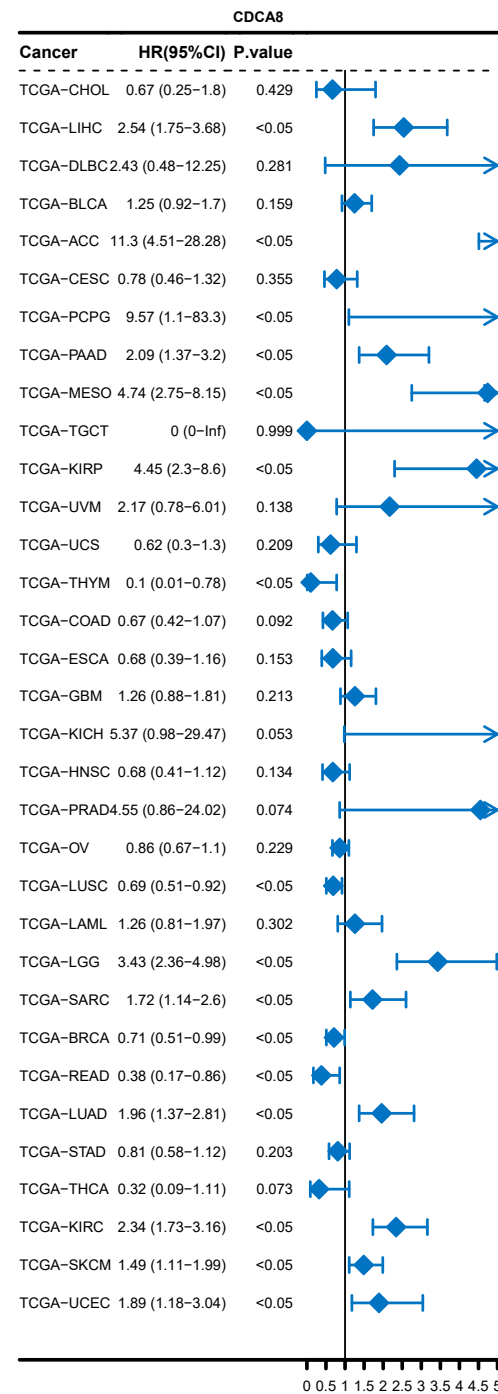

Supplement: Supplementary file 4 [file Image3.pdf]

A

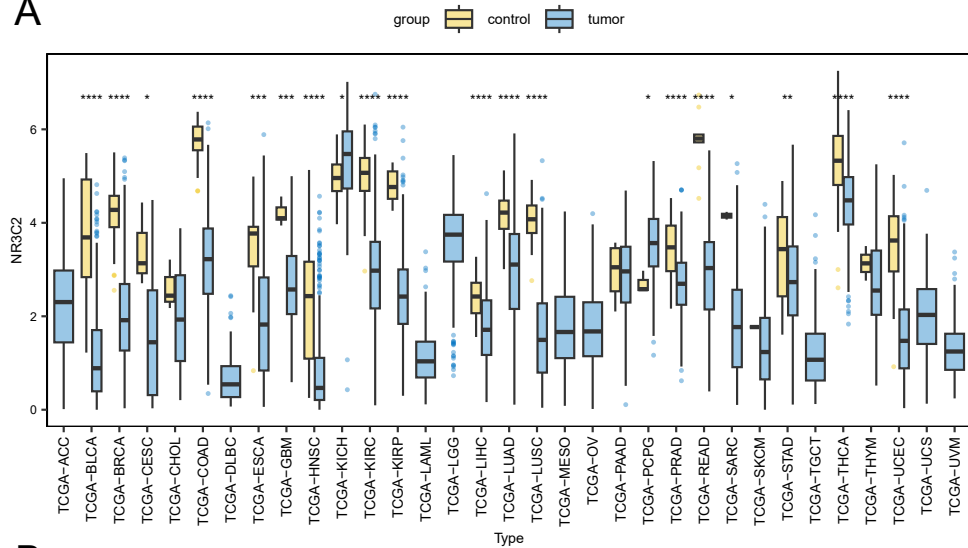

B

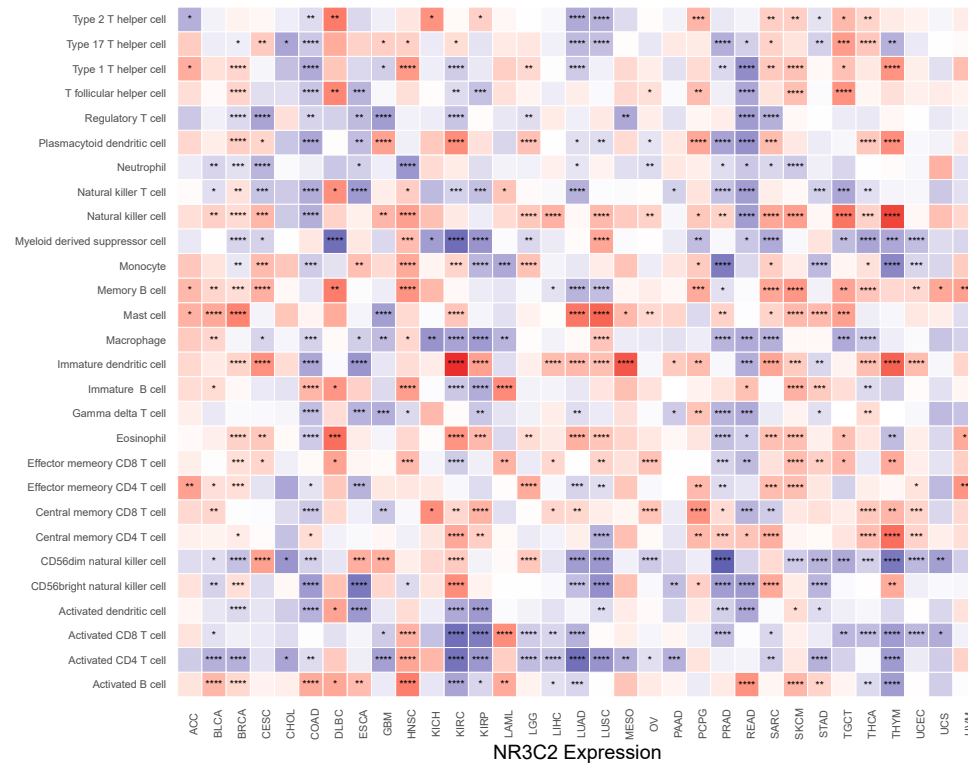

C

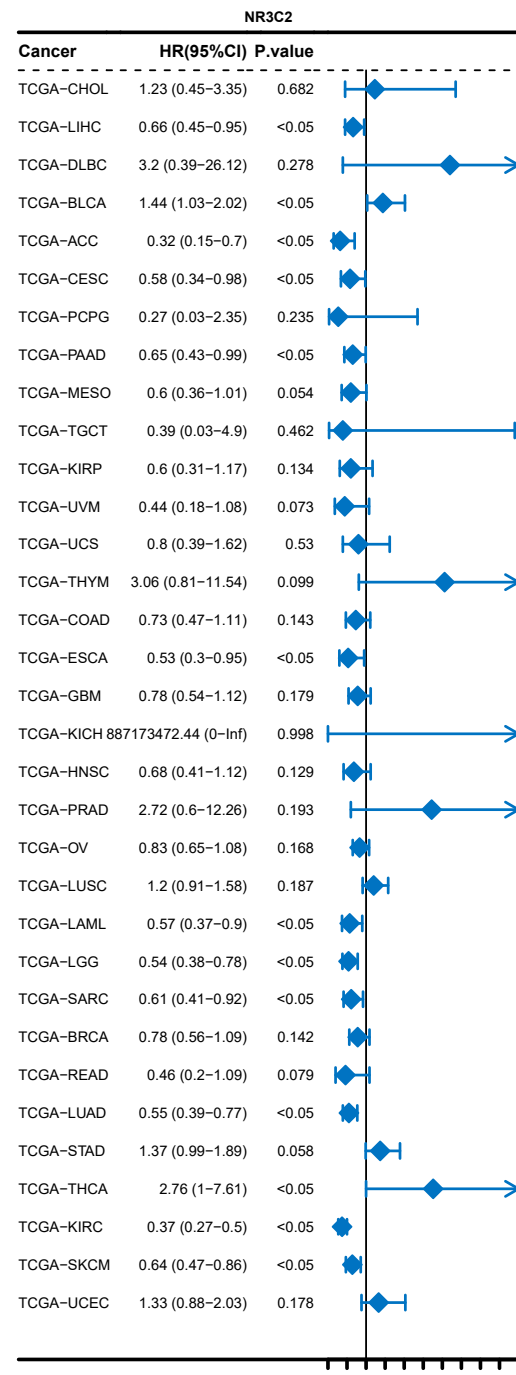

Supplement: Supplementary file 5 [file Image4.pdf]

A

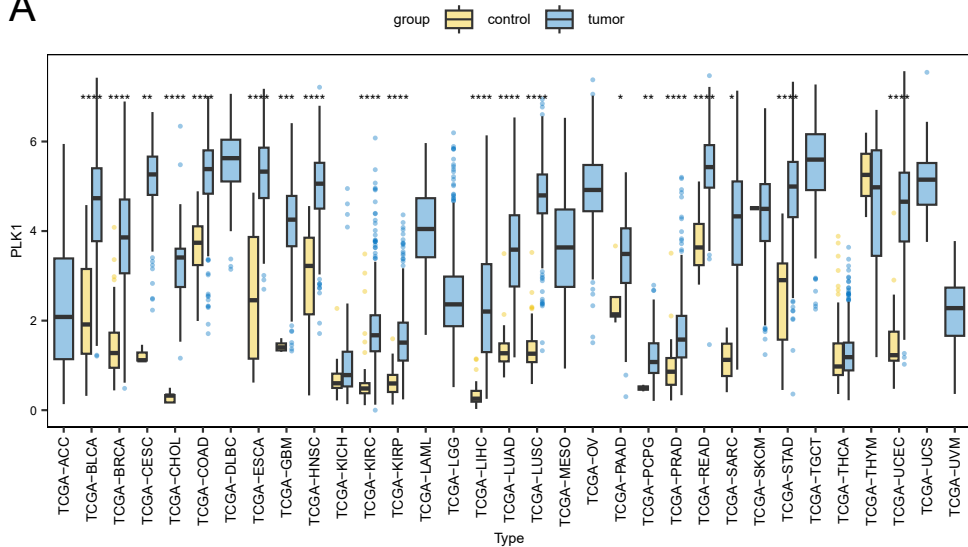

B

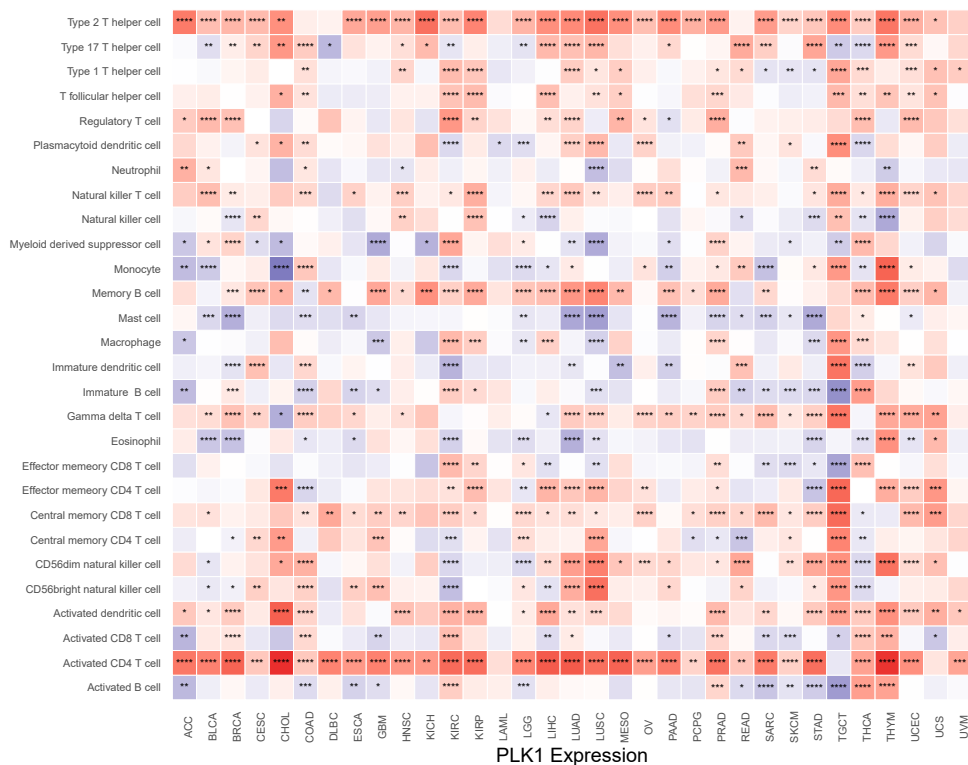

C

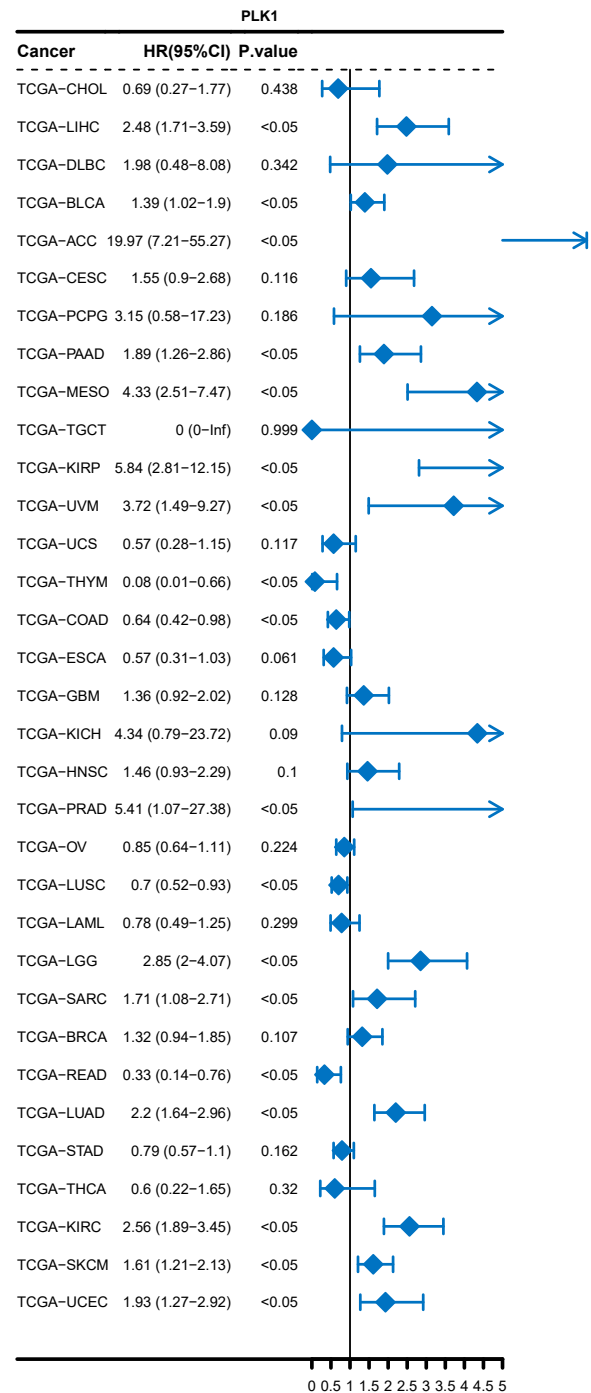

Supplement: Supplementary file 6 [file Image5.pdf]
